# Dual Roles of Ubiquitin-Specific Peptidase 10 (USP10) in Cancer

**DOI:** 10.3390/cells15060518

**Published:** 2026-03-13

**Authors:** Yifei Zhai, Liming Zhou, Manhan Zhao, Qiong Lin

**Affiliations:** School of Medicine, Jiangsu University, 301 Xuefu Road, Zhenjiang 212013, China; 2212413005@stmail.ujs.edu.cn (Y.Z.); 2212313098@stmail.ujs.edu.cn (L.Z.); 2212313096@stmail.ujs.edu.cn (M.Z.)

**Keywords:** USP10, deubiquitination, cancers, USP10 inhibitors, cancer therapy

## Abstract

Ubiquitin-specific peptidase 10 (USP10) deubiquitinates multiple signaling proteins in cancer cells. These USP10 substrates contain both tumor suppressors and oncogenic proteins, thus conferring both inhibitory and promoting effects of USP10 on tumorigenesis and progression. This review focuses on the dual roles of USP10 in various cancer types and addresses the association of aberrant USP10 expression with the development of various types of cancers, including hepatocellular carcinoma, lung cancer, breast cancer, prostate cancer, gastric cancer, and acute and chronic myelogenous leukemia. In addition, this review discusses the potential applications of USP10 inhibitors as targeted drugs for cancer therapy.

## 1. Overview of Deubiquitinating Enzymes (DUBs) and Ubiquitin-Specific Peptidases (USPs)

Deubiquitination is a reversible biochemical process of removing ubiquitin chains from ubiquitinated proteins that is catalyzed by DUBs. Deubiquitination plays a pivotal role in maintaining cellular homeostasis by regulating protein stability, interaction, activity, and subcellular localization [1,2,3]. DUBs function in multiple cellular processes, including gene expression, DNA repair, cell cycle, and apoptosis [4].

There are more than 100 DUBs found in human cells. DUBs are categorized into seven groups based on their sequence and structural domains, including ubiquitin-specific peptidases (USPs), ubiquitin carboxy-terminal hydrolases (UCHs), ovarian tumor proteases (OTUs), Machado–Joseph disease proteases (MJDs), novel miu-containing DUB family (MINDY) proteases, Jab1/MPN/MOV34 metalloenzymes (JAMMs), and Znfinger and UFSP structural domain proteins (ZUFSPs) [5,6,7].

The USP family is the largest DUB group, with more than 50 members. The USP members have a conserved USP domain comprising three regions: the deubiquitination catalytic region, the ubiquitin-binding region, and the substrate-binding region. These three regions of the USP domain are analogized to the finger, thumb, and palm, and have cysteine protease activity [8,9]. The USP catalytic region contains cysteine, histidine, and aspartate or glutamate residues that are responsible for hydrolyzing the ubiquitin chain [10]. Members of the USP family have variable sizes of USP domains that range from 300 to 800 amino acids [4]. USP members interact with the ubiquitin chain via blocking loops or specific ubiquitin-binding hotspots, combined with other protein-interactive domains, to determine substrate specificity [11]. Previous studies have shown that the auxiliary domains of USPs, such as ubiquitin-associated domains (UBA), ubiquitin-interaction motifs (UIM), and zinc-finger ubiquitin-specific protease domains (ZnF-UBP), enhance binding to ubiquitinated substrates [12].

USP family members play dual roles in cancers by inhibiting the degradation of either tumor suppressors or oncogenic proteins [13]. In addition, USP family members promote immune evasion by interfering with antigen processing and presentation [14].

In this review, we will address the dual roles of USP10, a well-studied USP family member, in cellular signaling and cancers, and discuss the potential application of USP10 inhibitors for cancer therapy.

## 2. USP10 and Its Cellular Function

### 2.1. The Deubiquitinase-Dependent Cellular Function of USP10

**Figure 1:**
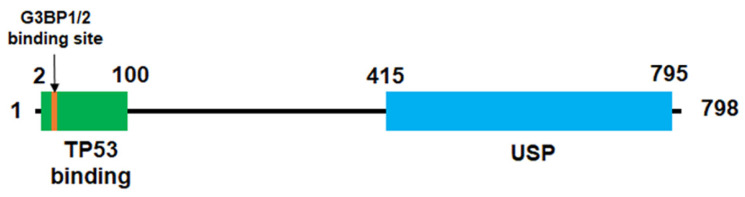
The domain structure of USP10. USP10 consists of 798 amino acids and has a conserved USP domain (aa 414-795) with ubiquitin carboxyl hydrolase (UCH) activity, a TP53-binding domain (aa 2-100), and a G3BP1/G3BP2-binding region (aa 6-21).

The human USP10 gene contains 18 exons and is located in band 1 of region 24 (16q24.1) on the long arm of chromosome 16 [1,15]. The USP10 protein consists of 798 amino acids with a conserved USP domain that has ubiquitin carboxyl hydrolase (UCH) activity, a TP53-binding domain, and a G3BP1/G3BP2-binding region (Figure 1). The USP10 protein is highly conserved both structurally and functionally from yeast to mammals, suggesting that USP10 is essential for cellular function.

**Figure 2:**
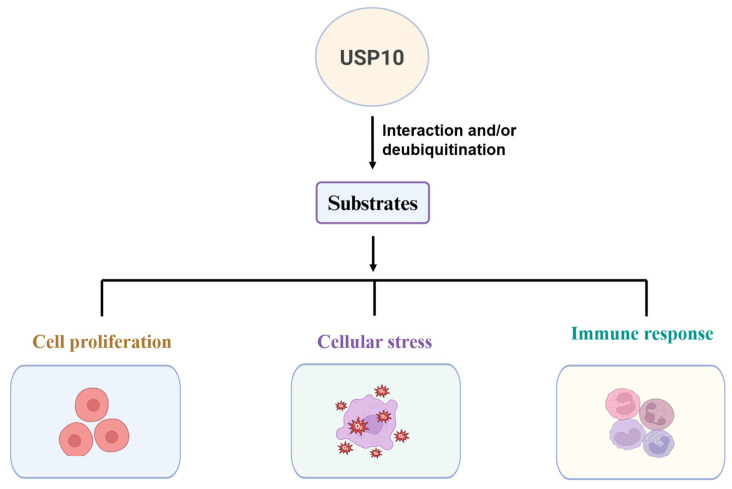
USP10 has multiple cellular functions by interacting with and deubiquitinating various substrates that regulate cell proliferation, cellular stress, and immune responses.

USP10 has a wide range of cellular functions involved in cell proliferation, cellular stress reactions, and immune response, contingent on its substrates (Figure 2). Aberrant USP10 function leads to pathological processes such as tumorigenesis, metabolic diseases, and neurodegenerative diseases.

USP10 regulates cell proliferation by interacting and deubiquitinating proteins involved in cell proliferation signaling, such as tumor suppressors, cell cycle proteins, and oncogenic proteins [16,17,18,19,20]. A number of proteins that regulate cell proliferation have been identified as USP10 substrates (Table 1). As these substrate proteins contain both tumor suppressors and pro-oncogenic proteins, USP10 functions in both inhibiting and promoting cell proliferation in a context-dependent manner. For example, the USP10 substrate TP53 inhibits cell proliferation in its wild-type form while promoting cell growth upon gain-of-function mutation. Accordingly, USP10 functions as a tumor suppressor in cells expressing wild-type TP53, whereas it acts as a pro-oncogenic protein in cells expressing mutant TP53 [21,22].

USP10 also participates in regulating cellular stress responses, including oxidative stress, nutritional stress, and endoplasmic reticulum (ER) stress [23,24,25]. USP10 deubiquitinates and stabilizes multiple stress-signaling proteins, including G3BP1/2, AMPK, PLAGL2, GPX4, FOXQ1, and Sirt6 [26,27,28,29,30,31,32,33], thereby regulating many cellular stress processes.

USP10 plays an important role in formation of stress granules, a type of membraneless cellular organelles containing RNA and proteins. Stress granules are induced by various stress conditions and involved in anti-stress and translational quality control in cells [34,35,36]. G3BP1 and 2 are the stress granule proteins and essential for stress granule formation [26,37]. It has been observed that USP10 interacts with G3BP proteins and inhibits the stress granule formation [37,38]. Furthermore, USP10 interacts with G3BP proteins to deubiquitinate ribosomal 40S subunits and inhibit their lysosomal degradation [39].

USP10 also regulates autophagy and lipid metabolism under stress conditions. USP10 ameliorates lipotoxicity by stabilizing Unc-51 like autophagy activating kinase 1 (ULK1) or activating autophagy-related signals to promote autophagy for removing damaged organelles or lipid droplets [40,41]. It has been observed that USP10 deubiquitinates the autophagy proteins Beclin1 and LC3 [42,43], thus is directly involved in regulation of autophagic process, suggesting that USP10 may directly participate in regulation of nutritional stress.

USP10 also participates in the hypoxic stress signaling pathway in tumor cells. Under hypoxic conditions, USP10 knockout in human colon cancer cells significantly enhanced the hypoxia-inducible factor 1-alph(HIF-1α) protein level. It seems that the effect of USP10 knockout on the HIF-1α protein level under hypoxic condition is mediated by activation of the mTOR/S6K-dependent protein synthesis [44]. In breast cancer cells, the deubiquitinase activity of USP10 is regulated by the hypoxia-induced non-coding RNA circWSB1. The circWSB1 directly binds to and sequesters USP10, which leads to TP53 degradation, thereby inhibiting apoptosis and promoting tumor cell proliferation and metastasis [45].

USP10 fulfills its function in the immune system mainly by regulating the stability of immune-related proteins through deubiquitination. These immune-related proteins are listed in Table 1. The major discoveries about the role of USP10 in regulation of cell immunity are related to the immune escape of tumor cells. USP10 deubiquitinates and stabilizes PD-L1 in tumor cells, thus enables tumor cells to avoid immune attack [46,47]. USP10 also stabilizes the YAP1/Cyr61 axis, thereby increasing M2 macrophage polarization and creating an immunosuppressive tumor microenvironment [48]. USP10 can promote M2 polarization by stabilizing the NLRP7 protein and further inhibiting anti-tumor immune responses [48,49,50]. A pan-cancer analysis revealed that high USP10 expression is associated with the increased infiltration of specific immune cells, such as regulatory T cells and M2 macrophages. These cells suppress the function of effector T cells by secreting immunosuppressive factors, such as IL-10 and TGF-β [16,50]. In hepatocellular carcinoma, USP10 has been shown to stabilize the SMAD4 and enhance the TGF-β signaling pathway, thereby promoting tumor metastasis and immune evasion [16,51]. In addition, USP10 deubiquitinates and stabilizes the transcription factor T-bet in immune cells, thereby promoting Th1 cell differentiation and IFN-γ production to counteract Th2-dominant inflammation [52].

In pancreatic adenocarcinoma and hepatocellular carcinoma, USP10 expression is correlated with the infiltration levels of specific immune cells and patient survival. Its absence results in reduced immune cell infiltration and apoptosis, thereby weakening antitumor immunity [50]. The effects of USP10 on the immune system are heterogeneous and sometimes even contradictory across cell types and microenvironments, or across disease stages, depending on the function of its substrates in the immune system.

**Table 1:** Substrates of USP10 involved in cell proliferation, immune responses, and oxidative stress.

Cellular Function Category	USP10 Function	Substrate or Interactive Protein	Mechanism
Cell proliferation	Promote cell proliferation	ANLN	USP10 deubiquitinates ANLN and maintains its stability, directly promoting cell division and proliferation [53].
		AR	USP10 deubiquitinates and stabilizes AR-associated target proteins, enhancing the efficiency of the AR signaling pathway, promoting the expression of androgen-responsive genes, and ultimately driving cell proliferation [26].
		ATMIN	USP10 deubiquitinates and stabilizes the ATMIN protein, and high ATMIN expression promotes proliferation and drug resistance in NPC cells [54].
		BAZ1A	USP10 stabilizes the BAZ1A protein through deubiquitination, forming a BAZ1A-SOX2-BRD4 complex that activates CSC signaling, upregulates expression of stemness-related genes, and drives cell proliferation [55].
		CCND1	USP10 promotes CCND1 stability by inhibiting its K48-linked polyubiquitination, thereby directly driving GBM cell proliferation [56].
		CD44	USP10 interacts with CD44, enhancing its binding to cytoskeletal proteins and activating the PDGFRβ/STAT3 signaling pathway, thereby promoting breast cancer cell proliferation [57].
		EIF4G1	USP10 deubiquitinates and stabilizes EIF4G1, thereby positively regulating its oncogenic function [58].
		ERα	USP10 stabilizes ESR1, thereby activating downstream proliferation signaling pathways to promote cell growth and division [59].
		FASN	USP10 deubiquitinates and stabilizes FASN, thereby promoting lipid synthesis and tumor cell proliferation [60].
		FLT3	In AML cells, USP10-mediated stabilization of FLT3 persistently activates downstream proliferation signaling pathways, leading to abnormal cell proliferation [61].
		FOXC1	USP10 activates the WNT signaling pathway by attenuating FOXC1 protein degradation, thereby promoting PDAC progression [62].
		G3BP1	USP10 interacts with G3BP1, inhibiting its degradation, thereby enhancing cellular resistance to chemotherapeutic drugs and promoting metastatic potential [63].
		G3BP2	In prostate cancer, USP10 stabilizes G3BP2 via deubiquitination, leading to TP53 translocation from the nucleus to the cytoplasm. This process inhibits TP53’s transcriptional activity and its subsequent mediation of cell cycle arrest and apoptosis [26].
		GRP78	USP10 stabilizes GRP78 protein levels and inhibits endoplasmic reticulum stress-mediated apoptosis to promote tumor cell proliferation [64].
		GSK3β	USP10 interacts with GSK3β (glycogen synthase kinase 3β) through deubiquitination, enhancing ULK1 transcription and promoting autophagy, proliferation, and invasion [40].
		HDAC6	In TP53-wild-typeNon-Small Cell Lung Cancer(NSCLC), the USP10-HDAC6 axis maintains proliferative capacity by deubiquitinating and stabilizing the oncogenic protein HDAC6, thereby helping cancer cells resist chemotherapy by inhibiting apoptosis [21].
		HDAC7	USP10 deubiquitinates HDAC7, enhancing its protein stability. HDAC7 reduces the acetylation and phosphorylation levels of β-catenin, promoting its transport from the cytoplasm to the nucleus. Nuclear β-catenin binds to the TCF4/LEF transcription factor, activating the expression of the downstream target gene FGF18, thereby promoting cell proliferation [65].
		IGF2BP1	USP10 stabilizes IGF2BP1 through deubiquitination, thereby promoting breast cancer metastasis [66].
		IGF2BP3	IGF2BP3 directly binds USP10 and impairs its ability to stabilize TP53, thereby promoting tumor proliferation [65].
		KLF4	USP10 deubiquitinates and stabilizes KLF4, thereby activating the TIMP3 tumor suppressor pathway and inhibiting cell proliferation and tumorigenesis in lung cancer [65].
		METTL13	USP10 directly binds to METTL13, inhibits its proteasomal degradation pathway by removing ubiquitin chains from the METTL13 protein, catalyzes m6A modification of downstream target genes, regulates CD44 mRNA degradation, activates the pSTAT3/PI3K–AKT signaling pathway, thereby enhancing chemotherapy resistance in CRPC [67].
		MOF	USP10 binds to MOF and deubiquitinates it, enhancing H4K16 enrichment at the ANXA2 promoter. This activates ANXA2 expression, further promoting transcriptional activity in the Wnt/β-catenin signaling pathway and accelerating ESCC progression [68].
		MSI2	USP10 regulates the stability of the oncogene MSI2 through deubiquitination, thereby promoting the proliferation of colon cancer cells [69].
		NICD1	USP10 regulates Notch signaling in endothelial cells by interacting with NICD1 to slow its ubiquitin-dependent degradation, thereby promoting pancreatic cancer proliferation [70,71].
		NLRP7	USP10 deubiquitinates and stabilizes NLRP7 protein, enhancing its pro-proliferative function in CRC [49].
		PABPC1	USP10 deubiquitinates and stabilizes PABPC1, enhancing its ability to bind mRNA and translational factors, thereby activating pro-proliferative signaling pathways and driving tumor cell proliferation [72].
		PCNA	USP10 can induce PCNA deubiquitination and promote the growth of HCC cells [20].
		PLAGL2	Adrenaline promotes HCC progression by upregulating USP10 through the ADRB2-c-Myc axis to stabilize PLAGL2 [27].
		PLK1	USP10 deubiquitinates PLK1, enhancing its stability and preventing proteasomal degradation. This activates downstream signaling pathways, thereby driving tumor cell proliferation [73].
		RAF1	USP10 stabilizes RAF1 protein levels by deubiquitinating it, thereby activating the RAF/MEK/ERK pathway and promoting cell proliferation [74].
		RCF2	In gastric cancer cells, USP10 significantly enhances cell migration and invasion capabilities by stabilizing RCF2 expression [75].
		RUNX1	In GBM, USP10 deubiquitinates and stabilizes RUNX1 levels, thereby promoting tumor cell proliferation [76].
		SKP2	USP10 directly binds to SKP2, thereby inhibiting its proteasomal degradation. The stabilization of SKP2 activates the Bcr-Abl signaling pathway, driving the proliferation of leukemia cells [77].
		SMAD4	USP10 enhances TGF-β signaling by deubiquitinating and stabilizing SMAD4, thereby promoting HCC proliferation [51].
		SNAI1	USP10 promotes survival and proliferation of intrahepatic cholangiocarcinoma cells by deubiquitinating SNAI1 [78].
		SSRP1	USP10 mediates deubiquitination, stabilizes SSRP1, and promotes MM cell proliferation [79].
		SYK	USP10 stabilizes the signal transduction protein SYK, thereby activating downstream proliferation-related pathways and enhancing cell proliferation, migration, and survival. Inhibiting USP10 degrades SYK, thereby suppressing SYK-dependent proliferation of leukemia cells [80].
		TCF4	USP10 deubiquitinates TCF4, promoting its protein stability and driving TNBC development and proliferation [17].
		TRAF4	USP10 interacts with TRAF4 to inhibit the TP53 pathway, leading to TP53 instability and thereby promoting cell proliferation and survival [81].
		XAB2	In CRC, USP10 stabilizes XAB2 through deubiquitination, thereby promoting ANXA2 transcriptional upregulation and enhancing proliferation, DNA damage repair, and oxaliplatin resistance [82].
		YAP/TAZ	USP10 promotes hepatocellular carcinoma proliferation by reversing the ubiquitination of YAP/TAZ [83].
		YBX1	USP10 deubiquitinates and stabilizes YBX1, thereby inhibiting PANoptosis and promoting oxaliplatin chemoresistance in gastric cancer cells [84].
		Yki	Usp10 enhances the Hippo signaling pathway by deubiquitinating and stabilizing Yki, thereby promoting cell proliferation [85].
	Suppress cell proliferation	ALK	USP10 reduces ALK protein stability through deubiquitination, thereby exerting a negative regulatory effect on ALK-mediated cell proliferation and metastasis processes [86].
		AMPKα	USP10 deubiquitinatesAMP-activated protein kinase catalytic subunit alpha (AMPKα), promoting its phosphorylation and amplifying activation. Phosphorylated AMPKα forms a positive feedback loop with USP10, inhibiting cell proliferation [87].
		AXIN1	USP10 enhances AXIN1-mediated β-catenin degradation, thereby suppressing excessive activation of the Wnt/β-catenin signaling pathway and inhibiting proliferation of colorectal cancer cells [88].
		CCND3	USP10 inhibits K48-linked polyubiquitination of CCND3, thereby stabilizing its activity, activating the CCND3/CDK4/6 signaling pathway, and inducing apoptosis [89].
		DIRAS2	USP10 deubiquitinates and stabilizes DIRAS2, thereby inhibiting the growth of pancreatic cancer cells [90].
		MSH2	During cell proliferation, USP10 deubiquitinates and stabilizes MSH2, thereby maintaining its DNA repair function. This helps prevent the accumulation of DNA damage and inhibits abnormal proliferation [91].
		TP53	USP10 inhibits tumor cell growth in cells harboring wild-type TP53, whereas it may promote tumor cell growth in the presence of TP53 mutations [92,93].
		PTEN	USP10 restores PTEN activity and inhibits proliferation in NSCLC cells [94].
		TNFRSF10B	USP10 inhibits epithelial–mesenchymal transition (EMT) by enhancing the stability of the TNFRSF10B protein, thereby suppressing the migration and invasion of gastric cancer cells [95].
		YTHDF2	YTHDF2 is a tumor suppressor factor. USP10 stabilizes YTHDF2 through deubiquitination, ultimately inhibiting melanoma cell proliferation [96].
		ZEB1	USP10 promotes the proteasomal degradation of ZEB1, reducing ZEB1 protein levels and inhibiting ZEB1-mediated tumor cell migration and metastatic capacity [97].
Immune Response	Promote immune response	AID	USP10 stabilizes the AID protein via deubiquitination, thereby supporting the generation of antibody diversity and efficient immune responses in B cells [98].
		CD36	In macrophages, USP10 interacts with CD36 and stabilizes the CD36 protein by cleaving polyubiquitin chains on CD36, thereby promoting the recognition and uptake of oxidized low-density lipoprotein and facilitating foam cell formation [99].
		NEMO	USP10 promotes immune responses by positively regulating the NF-κB signaling pathway through stabilizing NEMO [100].
	Suppress the immune response	B7-H4	USP10 promotes tumor immune escape by stabilizing B7-H4 protein expression. Inhibiting USP10 enhances B7-H4 degradation, thereby increasing tumor immunogenicity and improving the tumor-killing efficacy of SG [101].
		CXCR4	USP10 deubiquitinates and stabilizes CXCR4 protein, enhancing CXCR4-mediated signaling pathways. This promotes the secretion of immunosuppressive factors by tumor cells, suppresses CD8+ T cell function, and induces macrophage polarization toward the M2 phenotype, ultimately weakening the antitumor immune response [102].
		FOXQ1	USP10 enhances FOXQ1 stability through deubiquitination, thereby mitigating LPS-induced apoptosis and inflammatory responses [28].
		MAVS	USP10 removes the non-anchored K63-linked ubiquitin chain from MAVS through deubiquitination, thereby inhibiting RIG-I-mediated MAVS aggregation and type I interferon production [103].
		NLRP7	USP10 binds to and enhances NLRP7 protein stability, activating the NF-κB signaling pathway. This leads to phosphorylation of the transcription factor p65 and its translocation to the cell nucleus. Transcription and secretion of the chemokine CCL2 are upregulated, recruiting monocytes and inducing their differentiation into tumor-associated macrophages (TAMs) [49].
		PD-L1	USP10 mediates PD-L1 deubiquitination, thereby suppressing CD8+T cell infiltration and function and promoting immune escape of tumor cells [46].
		Sirt6	USP10 suppresses hepatic steatosis, insulin resistance, and inflammation by interacting with Sirt6 [104].
		T-bet	In peripheral blood mononuclear cells from asthma patients, USP10 deubiquitinates and stabilizes T-bet expression, counteracting Th2-dominant inflammatory responses [105].
		TAK1	USP10 reduces the production of inflammatory mediators by inhibiting TAK1 phosphorylation, thereby blocking excessive activation of its downstream JNK and p38 MAPK signaling pathways [106].
		TRAF6	USP10 deubiquitinates and blocks TRAF6-mediated downstream signaling, thereby inhibiting NF-κB transcriptional activity and preventing excessive immune activation during TLR and IL-1R-triggered inflammatory responses [107].
		UBE2S	UBE2S interacts with USP10 to enhance GLUT1 stability and promote glycolysis, thereby inducing M2 macrophage polarization, leading to TGF-β1 secretion and establishing an immunosuppressive microenvironment [108].
		YAP1	USP10 stabilizes YAP1 through deubiquitination, thereby upregulating the expression of the immunosuppressive factors PD-L1 and Galectin-9, thereby helping tumor cells evade immune attack [48].
Oxidative Stress	Promote oxidative stress	Tax	In HTLV-1-infected T cells, the Tax protein interacts with USP10, inhibiting USP10-mediated formation of peroxisome-like granules (SGs), promoting reactive oxygen species (ROS) production, and enhancing ROS-dependent apoptosis [109].
	Suppress oxidative stress	FOXO1	USP10 deubiquitinates and stabilizes FOXO1 protein. The stabilized FOXO1 translocates to the cell nucleus, activates antioxidant genes, and ultimately alleviates oxidative stress [110].
		FOXQ1	USP10 regulates FOXQ1 by stabilizing the FOXQ1 protein to suppress oxidative stress [28].
		G3BP1	Under oxidative stress conditions, USP10 is recruited to stress granules where it synergistically interacts with proteins such as G3BP1 to inhibit reactive oxygen species (ROS) accumulation and prevent apoptosis [111].
		Ku70/80	During chemotherapy, USP10 stabilizes the Ku70/80 complex in colorectal cancer, promotes DNA repair, reduces intracellular reactive oxygen species levels, and mitigates panapoptosis, thereby enhancing chemotherapy resistance [112].
		Nrf2	USP10 protein reduces dopamine-induced ROS production and ROS-dependent apoptosis in neurons by stimulating Nrf2 antioxidant activity [113].
		p62	USP10 synergizes with p62 to induce aggregation of ubiquitinated proteins, inhibit proteasome activity, and reduce the toxic accumulation of ubiquitinated proteins, thereby suppressing reactive oxygen species-dependent apoptosis [114].
		PARP1	USP10 deubiquitinates and stabilizes PARP1, ensuring that PARP1 can continuously perform its function of promoting the transcription of antioxidant enzymes [115].
		POLR2A	USP10 removes POLR2AK48- and K63-linked ubiquitin chains, enhancing stability and promoting transcription of the cystine/glutamate antiporter gene SLC7A11. This enhances cellular cystine uptake, promotes glutathione synthesis, and inhibits lipid peroxidation and ferroptosis [116].
		Sirt6	USP10 deubiquitinates and stabilizes Sirt6, promoting Nrf2 activation and its binding to the antioxidant response element (ARE). This upregulates the expression of downstream antioxidant genes, thereby reducing reactive oxygen species (ROS) accumulation and oxidative damage [23].

### 2.2. The Deubiquitinase-Independent Cellular Function of USP10

USP10 also regulates cellular processes through the non-deubiquitination mechanism. It was observed that USP10 functions as an antagonist of β-catenin activity by promoting phase separation, a process that is independent of its deubiquitination activity. USP10 can inhibit tumor development through this antagonizing β-catenin activity, both in a deubiquitinase-dependent and -independent manner [88]. Upon DNA damage, USP10 undergoes nuclear translocation. Within the nucleus, USP10 achieves its regulatory function by both deubiquitinase and non-deubiquitinase activities. It stabilizes nuclear TP53 by deubiquitination to induce cell cycle arrest, while simultaneously disrupts β-catenin transcription complexes by mediating phase separation through its intrinsically disordered region (IDR), thereby non-catalytically blocking oncogenic signaling pathways [88,117]. USP10 also binds to transmembrane transporter proteins or protein complexes, thereby facilitating the transfer of biomolecules across membranes [118].

Currently, the non-deubiquitinase-dependent cellular function of USP10 is still not fully characterized, and its significance in cell regulation remains undefined.

## 3. Cellular Signaling Pathways Regulated by USP10

**Figure 3:**
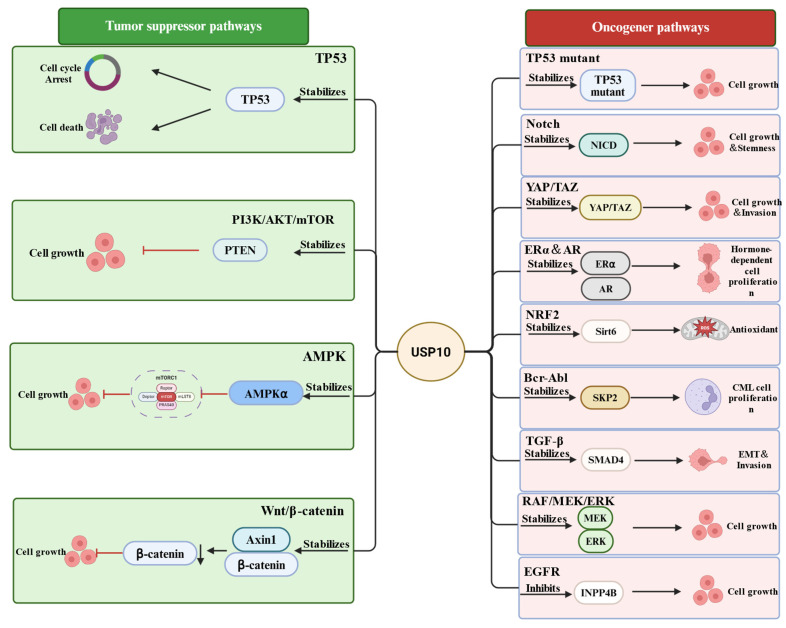
USP10 plays dual roles in cancers dependent on the substrate-mediated tumor suppressing or promoting signaling pathway.

By interacting with and deubiquitinating diverse cellular signaling proteins, USP10 regulates multiple signaling pathways (Table 2 and Figure 3). These signaling pathways are involved in many pathological processes, including tumorigenesis, metabolic and neuronal degenerative diseases. Here, we focus on the signaling pathways involved in tumorigenesis.

As mentioned above, USP10 has dual roles in tumorigenesis contingent on the function of its substrates in cancer. Accordingly, the cellular signaling pathways regulated by USP10 are divided into two categories: suppressing and promoting tumorigenesis.

### 3.1. Tumor Suppressing Signaling Pathways Regulated by USP10

It has been observed that USP10 plays a tumor suppressing role mainly through regulating four tumor suppressing signaling pathways. These signaling pathways include the TP53 tumor suppressing signaling pathway; the PTEN tumor suppressing signaling pathway; the AMPK signaling pathway; and the Wnt/b-catenin down-regulation pathway.

#### 3.1.1. The TP53 Tumor Suppressing Signaling Pathway

TP53, a pivotal tumor suppressor gene that is frequently mutated in various cancers, plays a crucial role in suppressing cancer by impeding the proliferation of damaged cells in response to stress [122]. Under normal conditions, USP10 localizes in the cytoplasm and directly binds to the MDM2-ubiquitinated TP53, removes the K48 ubiquitin chain that stabilizes TP53, and allows TP53 to continue its function. In the event of DNA damage, USP10 is phosphorylated by ATM kinase and translocated to the nucleus. The nuclear USP10 enhances TP53 transcription activity and promotes the transcription of the TP53 target genes p21, BAX, and PUMA. This, in turn, triggers cell cycle arrest or apoptosis [92].

#### 3.1.2. The PTEN Tumor Suppressing Signaling Pathway

USP10 stabilizes PTEN by removing the K63 ubiquitin chain [94,119]. The primary function of deubiquitinated PTEN as a pivotal anti-oncogenic factor is to dephosphorylate phosphatidylinositol-3,4,5-trisphosphate (PIP3) to phosphatidylinositol-4,5-bisphosphate (PIP2). This process reduces PIP3 levels, thereby impairing PDK1 and AKT co-localization at the cellular membrane and inactivating AKT [123,124].

#### 3.1.3. The AMPK Signaling Pathway

USP10 has been shown to inhibit the polyubiquitination of AMPKα, thereby reducing its degradation and promoting phosphorylation at key sites, thereby enhancing AMPK kinase activity. It has been demonstrated that the AMPK substrate exerts an inhibitory effect on the downstream mTORC1 signaling pathway. Consequently, this results in the suppression of protein synthesis, cell proliferation, and survival [87,119,125].

#### 3.1.4. The Wnt/β-Catenin Down-Regulation Pathway

USP10 has been shown to bind to AXIN1 via a conserved motif and remove its K48-linked ubiquitin chain, thereby preventing AXIN1 from the proteasome-mediated degradation. This results in β-catenin degradation, thereby inhibiting the β-catenin-dependent gene transcription and suppressing the β-catenin-mediated tumorigenesis [88]. In lung cancer, USP10 has been observed to bind directly to PTEN, thereby stabilizing PTEN through deubiquitination. PTEN inhibits the PI3K/AKT pathway, which causes cross-inhibition of Wnt/β-catenin signaling and suppression of tumor growth and invasion [126].

### 3.2. Tumor Promoting Signaling Pathways

#### 3.2.1. The TP53 Mutant Oncogenic Signaling Pathway

The mutant TP53 protein has been shown to participate in and promote multiple oncogenic signaling pathways through its acquired functions, thereby driving malignant tumor progression [127].

In TP53-mutated cancers, USP10 exhibits oncogenic properties. USP10 facilitates tumor cell survival, proliferation, and chemotherapy resistance by deubiquitinating and stabilizing oncoproteins, such as HDAC6, or by interacting with circRNAs to enhance mutant TP53 activity [21,25]. Under specific conditions, USP10 can also synergistically promote tumor progression by regulating pathways that interact with TP53 mutants, such as YAP/TAZ and Wnt/β-catenin [25,128].

In cancers harboring TP53 mutations, USP10 undergoes an oncogenic transformation, shifting from its typical role as a tumor suppressor to that of an oncogenic co-activator. This role reversal positions USP10 as a highly promising therapeutic target. Inhibiting USP10 reduces the stability of oncogenic mutant TP53 proteins, and thus has potential for effectively blocking multiple tumor-promoting signaling pathways driven by the TP53 mutants.

#### 3.2.2. The Notch Signaling Pathway

The Notch signaling pathway has been identified as a critical oncogenic pathway in various tumors. The core mechanism of this process involves the abnormal activation of the Notch receptor, which results in the persistent presence of its intracellular domain (NICD) and thereby sustains prolonged high activity in downstream transcriptional networks. USP10 interacts with NICD and antagonizes theubiquitination-mediated degradation of NICD1. The stabilization of NICD1 protein is a critical step that enables sustained activation of the Notch signaling pathway and enhances transcription of its downstream oncogenes. USP10 enhances Notch signaling by stabilizing NICD1 in multiple types of cancer [70]. This aberrant Notch signaling displays oncogenic effects, fostering tumor cell proliferation, survival, invasion, metastasis, and perpetuation of tumor stem cell properties [129,130,131]. Inactivation of USP10 reduces the NICD1 level, weakens Notch signaling, reduces tumor cell growth rate, and enhances cancer cells’ sensitivity to chemotherapy [70,71]. USP10 forms a distinct oncogenic axis with the Notch signaling pathway. Targeting this oncogenic axis has potential to develop strategies to overcome progression and drug resistance in the Notch-associated tumors.

#### 3.2.3. The YAP/TAZ Signaling Pathway

The Hippo pathway is crucial for regulation of cell proliferation, differentiation, and migration. The YAP/TAZ signaling pathway is a core co-activator downstream of this pathway. Dysregulation of this pathway has been implicated in initiation, progression, and metastasis of various cancers. In the event of aberrant activation, YAP/TAZ translocate into the nucleus, thereby inducing expression of oncogenes. The process has been shown to promote tumor cell proliferation and survival, while suppressing apoptosis [132]. In hepatocellular carcinoma patient samples, USP10 expression is positively correlated with YAP/TAZ expression. Mechanistically, USP10 stabilizes YAP/TAZ proteins through a direct interaction, thereby inhibiting the proteasome-mediated degradation of YAP/TAZ. In contrast, USP10 deficiency accelerates the degradation of YAP/TAZ proteins via the ubiquitin-proteasome pathway and inhibits the proliferation of hepatocellular carcinoma cells in both in vivo and in vitro models [83]. Therefore, USP10 acts as a key upstream regulator in this oncogenic signaling pathway by deubiquitinating and stabilizing YAP/TAZ, thereby jointly promoting malignant tumor progression.

#### 3.2.4. The Estrogen Receptor Alpha (ERα) Signaling Pathway

ERα is a key driver of breast cancer progression, promoting cancer cell proliferation, migration, and survival by activating the transcription of its target oncogenic genes. In 70–80% of breast cancer cases, sustained activation of the ERα signaling pathway leads to tumor growth and resistance to endocrine therapy [133,134]. In breast cancer, USP10 plays a key regulatory role in the ERα signaling pathway by deubiquitinating and stabilizing ERα, thereby enhancing the ERαoncogenic signaling. The stabilization of ERα by USP10 has been shown to enhance the ERα transcriptional activity, thereby promoting breast cancer cell proliferation, migration, and tumor growth. This process is also regulated by upstream factors. For example, the oncogene ARL3 (ADP-ribosylating protein-like) can upregulate USP10 and form an ARL3-USP10-ERα axis that maintains ERα stability and oncogenic function [59].

#### 3.2.5. The Sirt6/Nrf2 Oxidative Stress Signaling Pathway

The role of the Sirt6/Nrf2 oxidative stress signaling pathway in promoting carcinogenesis primarily manifests through enhancing the antioxidant defense capabilities of cancer cells, thereby promoting tumor cell survival, proliferation, metastasis, and drug resistance. Specifically, Sirt6 activates the Nrf2 signaling pathway through deacetylation, thereby upregulating expression of antioxidant genes and reducing intracellular oxidative stress [135,136]. This signaling pathway protects cells from oxidative damage, thereby antagonizing the induction of apoptosis by oxidative stress. Furthermore, activated Sirt6 has been observed to interact directly with Nrf2, thereby reducing its binding to Keap1 and consequently inhibiting Nrf2 ubiquitination and degradation. This causes accumulation of Nrf2 protein within the nucleus and enhances the Nrf2 transcriptional activity, thus promotes cancer cell survival and proliferation [137,138].

USP10 functions in the Sirt6/Nrf2 pathway by binding directly to Sirt6, reducing ubiquitination and degradation of Sirt6, and sustaining activation of the Sirt6/Nrf2 pathway in cells [23,30,31]. In thyroid cancer, the USP10-Sirt6-Nrf2 axis has been demonstrated to promote the expression of glutathione peroxidase 4 (GPX4), thereby effectively inhibiting ferroptosis and enhancing cancer cell survival and malignant phenotypes [32].

The USP10-Sirt6-Nrf2 pathway is a highly promising therapeutic target. Interventions of this pathway may compromise the antioxidant barrier of cancer cells while protecting normal tissues, thereby enhancing the efficacy of existing therapies.

#### 3.2.6. The SKP2 Cell Cycle Signaling Pathway

SKP2 plays a pivotal role in carcinogenesis by regulating cell cycle progression. As a core component of the SCFE3 ubiquitin ligase complex, SKP2 functions in ubiquitination and degradation of cell cycle inhibitors p21 and p27. This process is pivotal in the release of the G1 checkpoint block and promotion of cell cycle transition from the G1 phase to the S phase [139]. In neoplastic cells, SKP2 promotes p27 degradation and facilitates the G1/S phase transition in the cell cycle in cancers such as gliomas and osteosarcomas [140]. In osteosarcoma, SKP2 not only promotes cell proliferation and invasion but also suppresses apoptosis [141]. Inhibition of SKP2 induces the G0/G1 phase arrest and tumor cell apoptosis [142]. In addition, inactivation of YAP inhibits SKP2 transcription, leading to accumulation of p21/p27 and cell cycle arrest [143].

USP10 has been observed to bind to and deubiquitinate SKP2, thus preventing SKP2 from proteasomal degradation and extending the half-life of SKP2 [144]. In Chronic Myelogenous Leukemia (CML), the USP10-mediated elevation of SKP2 not only enhances the degradation of cell cycle inhibitors such as p27, but also induces the K63-linked ubiquitination and activation of oncogene Bcr-Abl [77].

The USP10/ SKP2 cell cycle signaling pathway exerts oncogenic effects in multiple cancers. Thus, the USP10/SKP2 pathway is a potential target for cancer therapy.

#### 3.2.7. The TGF-β/SMAD Signaling Pathway

The TGF-β/SMAD signaling pathway primarily exerts tumor-suppressing effects in early-stage or normal cells. As tumor progresses, the TGF-β/SMAD signaling pathway transforms into a key oncogenic driver. EMT has been identified as a critical oncogenic process of the TGF-β/SMAD pathway, which leads to the loss of epithelial polarity. The TGF-β/SMAD signaling pathway-mediated EMT has been shown to enhance cancer cell migration and invasive capabilities, thereby promoting metastasis [145,146].

USP10 directly binds to and deubiquitinates SMAD4 protein, thereby preventing SMAD4 degradation. Therefore, USP10 stabilizes SMAD4 and enhances the TGF-β/SMAD4 signaling. As USP10 stabilizes SMAD4 and activates the TGF-β/SMAD pathway, it may be a potential therapeutic target for cancer treatment. USP10 inhibitors have been used to impede the TGF-β/SMAD signaling in liver cancer [51]. 

#### 3.2.8. The RAF1 Oncogenic Signaling Pathway

RAF kinase has been identified as a key protein in the RAS-RAF-MEK-ERK signaling pathway, playing a crucial role in regulation of various cellular processes, including cell proliferation, differentiation, and survival [147]. RAF1 is a direct downstream effector of RAS GTPase. Upon activation by upstream signals, RAF undergoes the MAPK cascade, which activates multiple transcription factors and transcription of cell proliferation and cell-cycle-related genes, thereby driving cancer cell proliferation and growth. In various cancers, overexpression or mutation of RAF1 leads to excessive activation of this MAPK pathway and promotes tumorigenesis [148,149].

USP10 has been shown to reduce the ubiquitination-mediated degradation of RAF1 through deubiquitination, thereby enhancing the RAF1 protein level and activating the RAF1/MAPK pathway. The USP10-mediated activation of the RAF1/MAPK pathway promotes cancer cell proliferation and migration, as well as suppresses apoptosis [74].

#### 3.2.9. The Androgen Receptor (AR) Signaling Pathway

It has been observed that USP10 enhances tumor progression by deubiquitinating and stabilizing AR protein, which in turn enhances the AR-mediated transcriptional activity and activates the downstream oncogenic gene expression. The AR signaling pathway promotes the proliferation and survival of prostate cancer cells and is closely associated with poor patient prognosis [26].

#### 3.2.10. EGFR Signaling Pathway

A recent study found that USP10 is involved in the EGFR signaling in colorectal cancer cells. Knockdown of USP10 in colorectal cancer cells significantly reduced sensitivity of the cells to inhibition by the EGFR inhibitors. Mechanistic studies found that the EGFR-mediated colorectal cancer cell proliferation is caused by activation of AKT, and this EGFR-mediated activation of AKT is negatively regulated by inositol polyphosphate 4-phosphatase type II B (INPP4B). The effect of USP10 on the EGFR-mediated colorectal cancer cell proliferation is not from direct interaction or deubiquitination of EGFR, but rather from direct enhancement of the activation of AKT by the USP10-dependent reduction in the INPP4B protein level. However, the mechanism underlying the USP10-dependent reduction in INPP4B protein level remains unclear. These findings have established the USP10/INPP4B/AKT axis in the EGFR signaling pathway in colorectal cancer [121].

## 4. The Cancers Regulated by USP10

Consistent with the dual roles in regulation of cellular signaling, USP10 plays dual roles in regulation of cancers. The cancers regulated by USP10 are summarized in Table 3. It should be noted that USP10 may have an opposite function for the same type of cancer, such as lung cancer or liver cancer, contingent on the function of its substrate and the associated signaling context. The following are the cancer types that are either suppressed or promoted by USP10.

### 4.1. The Cancers That Are Suppressed by USP10

#### 4.1.1. Lung Cancer

USP10 expression is repressed in lung cancer tissues, and the suppression of USP10 has been observed to promote lung cancer cell proliferation, invasion, and tumor growth in vivo [25]. Overexpression of USP10 leads to inhibition of AKT through direct binding to PTEN [94,126]. USP10 also inhibits degradation of the transcription factor KLF4 at the early stage of lung cancer and reduces the invasiveness and metastatic potential of tumor cells, thereby inhibiting the progression of lung cancer [152]. USP10 stabilizes TP53 to induce cell cycle arrest or apoptosis in response to DNA damage [92,153]. In non-small cell lung cancer (NSCLC), USP10 also stabilizes the tumor suppressor p14ARF through deubiquitination, thereby inhibiting cell proliferation and inducing cell senescence [154].

#### 4.1.2. Liver Cancer

In liver cancer, USP10 directly interacts with PTEN and AMPKα and stabilizes their protein levels by deubiquitination. The USP10-PTEN-AMPKα axis functions as a negative regulator of the mTOR signaling pathway and inhibits liver cancer cell proliferation [119]. USP10 also activates LKB1 protein in hepatocellular carcinoma cells by inhibition of the proteasome-mediated LKB1 degradation, which in turn activates the AMPK signaling pathway. The activation of the LKB1-AMPK pathway inhibits metabolic reprogram of tumors, thereby suppressing the proliferation of hepatocellular carcinoma cells. In clinical manifestations, expression of USP10 is positively correlated with that of LKB1. Liver cancer patients with high USP10 expression have a higher tumor differentiation level and prolonged overall survival [18]. In addition, USP10 promotes autophagy in hepatocellular carcinoma cells by activating the JNK1/TSC2 signaling pathway, thereby inhibiting cell steatosis and tumor progression [155].

#### 4.1.3. Colorectal Cancer

The majority of colorectal cancers are caused by aberrant activation of the Wnt/β-catenin signaling pathway [156]. It has been observed that USP10 disrupts the function of β-catenin by promoting the phase separation of β-catenin, thus inhibits the Wnt/β-catenin signaling and the growth of colorectal cancer cells [88]. Meanwhile, USP10 interacts with and stabilizes scaffold protein AXIN1, resulting in an increase in binding of AXIN1 to β-catenin and inhibition of the Wnt/β-catenin pathway, thus blocking the colorectal tumor progression [157]. USP10 inhibits colorectal tumor metastasis by directly binding to and deubiquitinating the transcription factor ZEB1 [97].

#### 4.1.4. Gastric Cancer

Low expression of USP10 in gastric cancer was positively correlated with tumor invasion depth, lymph node metastasis, and poor prognosis. These findings suggest that USP10 may play a tumor suppressor role by inhibiting tumor invasion and metastasis. Studies on the molecular mechanisms indicate that USP10 stabilizes TNFRSF10B via deubiquitination, thereby inhibiting the epithelial-mesenchymal transition (EMT), suggesting that inhibition of EMT is the mechanism by which USP10 exerts the tumor-suppressing function in gastric cancer [95].

#### 4.1.5. Some Other Cancers That Are Suppressed by USP10

In renal cell carcinoma, expression of USP10 is negatively associated with cancer progression and poor prognosis. Absence of USP10 promotes the ubiquitination and degradation of Sirt6 and TP53, which activates *c-Myc* and accelerates tumor development. Restoring USP10 expression stabilizes the tumor suppressor proteins, inhibits the transcriptional activity of the oncogene *c-Myc*, blocks cell cycle progression, and reduces tumor growth [92,150]. 

USP10 expression in thyroid cancer cells is lower than in normal thyroid cells, and further reduced in the drug-resistant thyroid cancer cells. Restoring USP10 expression inhibited the cancer cell invasion, migration, and epithelial–mesenchymal transition (EMT) while promoting cell apoptosis [151]. This effect is associated with inhibition of the PI3K/AKT pathway. Overexpression of USP10 reverses the Adriamycin resistance and reduces cancer cell migration, invasion, and EMT by inhibiting ABCG2 and PI3K/AKT signaling [151].

### 4.2. The Cancers That Are Promoted by USP10

#### 4.2.1. Prostate Cancer

In prostate tissue, USP10 is primarily cytoplasmic, and its expression positively correlates with AR expression. Consistently, high expression of USP10 is associated with poor prognosis of prostate cancer patients. G3BP2, a USP10 substrate, is upregulated upon AR activation, forming a positive feedback loop that promotes prostate tumor growth. USP10 interacts directly with G3BP2 and stabilizes G3BP2 through deubiquitination, thereby promoting prostate cancer cell proliferation and inhibiting TP53 nuclear translocation. This, in turn, impairs the TP53-mediated tumor suppressor signaling [26]. Knockdown of USP10 inhibits G3BP2-mediated pro-carcinogenic effect. 

#### 4.2.2. Breast Cancer

USP10 directly interacts with and stabilizes CD44, a key marker of breast cancer stem cells, through deubiquitination, thereby activating the PDGFRβ/STAT3 signaling pathway and promoting breast tumor stemness, metastasis, and chemoresistance [57]. USP10 also facilitates breast cancer metastasis by stabilizing IGF2BP1 through deubiquitination and activating the metabolic enzyme CPT1A [66]. USP10 promotes DNA damage repair in breast cancer cells by deubiquitinating the K418 site of PARP1 in an ATM-dependent manner, thereby stabilizing the PARP1 protein that leads to resistance to PARP1 inhibitors [158]. In APC gene-truncated breast cancer cells, USP10 binds to β-catenin through its unstructured N-terminus, antagonizes the APC-mediated β-catenin degradation, maintains β-catenin stability, and promotes the undifferentiated state and metastatic potential of the tumor [159].

#### 4.2.3. Pancreatic Ductal Adenocarcinoma

USP10 interacts with FOXC1, a transcription factor that plays a pivotal role in the progression of pancreatic ductal adenocarcinoma, and enhances FOXC1 stability through deubiquitination. In addition to this deubiquitination-mediated stabilization, USP10 also regulates FOXC1 stability through phosphorylation, promoting cancer cell proliferation [62]. USP10 has been shown to promote survival and invasion of pancreatic ductal carcinoma cells by attenuating endoplasmic reticulum (ER) stress. Knockdown of USP10 induces upregulation of BiP and ER stress, leading to activation of the UPR, which in turn inhibits pancreatic cancer cell viability, clone formation, and invasiveness. Consistently, USP10 protects tumor cells from ER stress and promotes tumor progression [24]. The USP10-Notch1 signaling axis is oncogenic in pancreatic cancer. USP10 stabilizes Notch1 and enhances its signaling, thereby supporting the survival and chemo-resistance of pancreatic cancer cells. The tumor suppressor N1DARP disrupts the interaction of USP10 with Notch1, thus promotes the degradation of Notch1 and inhibits progression of pancreatic cancer [71].

#### 4.2.4. Lung Cancer

Although USP10 has an inhibitory role in lung cancer progression by stabilizing the tumor suppressor PTEN as we discussed above, USP10 also promotes lung cancer by interaction with its oncogenic substrate proteins. Elevation of EIF4G1 through the USP10-mediated deubiquitination promotes proliferation, migration, and invasion of non-small cell lung cancer cells [58]. Mutations of TP53 frequently occur in lung cancer. Mutations of TP53 leads to loss of its tumor suppression function and gain of oncogenic function [160,161]. Stabilizing TP53 mutants promotes lung cancer progression. USP10 interacts with and stabilizes HDAC6. In TP53-mutated lung cancer cells, overexpression of USP10 or HDAC6 induces cisplatin resistance and forms the USP10-HDAC6-cisplatin resistance axis. This axis may play an important role in resisting the treatment of lung cancer [21].

#### 4.2.5. Liver Cancer

USP10 also has dual roles in hepatocellular carcinoma and is found to promote liver cancer progression. USP10 directly binds to the transcription factor SMAD4, cleaves the ubiquitin chain of SMAD4, and maintains its protein abundance. The TGF-β/SMAD4 signaling pathway has been identified as a key factor in promoting HCC metastasis [51,162]. Recent studies have identified a novel role of USP10 in promoting growth, migration, and invasion of hepatocellular carcinoma cells [27,51,83]. This promotion is associated with an increase in the metabolic levels of proline and hydroxyproline that leads to stabilization of the YAP1 protein and enhancement of activity of the YAP1/TEAD transcription factor complex [163].

#### 4.2.6. Leukemia

In acute myeloid leukemia (AML), the protein kinase SYK has been shown to promote leukemia progression synergistically with FLT3-ITD [164]. USP10 plays a pivotal role in promoting AML by directly binding to and deubiquitinating FLT3-ITD. Thus, USP10 prevents FLT3-ITD from proteasomal degradation and enhances the FLT3-ITD oncogenic signaling [61,165]. USP10 also deubiquitinates and stabilizes SYK, thus has the capacity to stabilize both SYK and FLT3-ITD, thereby establishing a positive feedback loop that amplifies oncogenic signaling and fosters proliferation of AML cancer cells [80]. USP10 also activates BCR-ABL fusion proteins by deubiquitinating and stabilizing SKP2 in CML (Chronic Myelogenous Leukemia). SKP2 can amplify the BCR-ABL oncogenic signal and directly promote proliferation and survival of CML cells [77].

## 5. USP10 Inhibitors for Cancer Treatment

**Figure 4:**
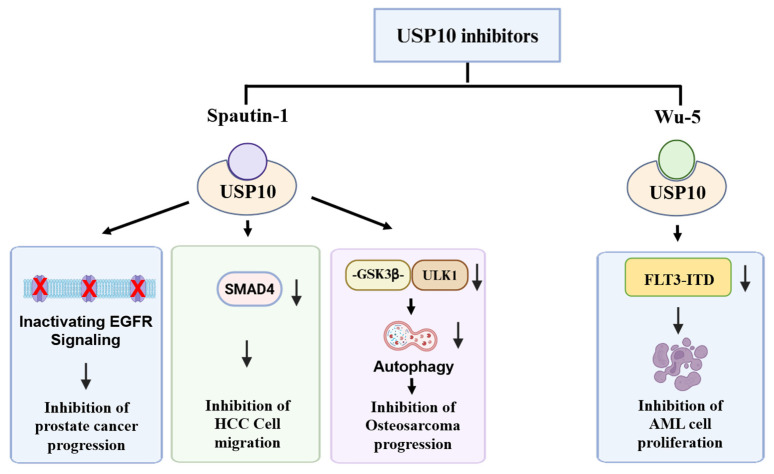
USP10 inhibitors, as potential drugs for cancer treatment, inhibit prostate cancer, liver cancer, bone cancer, and leukemia progression in preclinical studies. By inhibition of USP10, spautin-1 inactivates the EGFR signaling pathway to inhibit prostate cancer progression, induces SMAD4 degradation and impedes the SMAD4-mediated cell migration of HCC cells, and inhibits the GSK3β-ULK1 axis and cell autophagy to delay osteosarcoma progression. The other USP10 inhibitor Wu-5 promotes FLT3-ITD protein degradation, thus inhibits AML cell proliferation.

As the oncogenic substrates of USP10, such as TP53 mutants, play important roles in tumorigenesis and progression and are down-regulated by ubiquitination-mediated degradation, inhibition of USP10 has emerged as an important targeted cancer therapeutic strategy. Thus, USP10 inhibitors represent a class of small-molecule compounds for the development of anti-cancer drugs [166]. To date, the USP10 inhibitors spautin-1 and Wu-5 have been used in preclinical cancer research as summarized in Figure 4.

### 5.1. Spautin-1

Spautin-1 is a specific inhibitor of USP10 and has been demonstrated to inhibit cancer development. Spautin-1 exerts its anticancer effect by modulating the oncogenic signaling pathway. In prostate cancer, spautin-1 impairs activation of the epidermal growth factor receptor (EGFR) signaling pathway and induces cell cycle arrest and apoptosis [76,89,167]. In HCC, spautin-1 reduces the SMAD4 protein level upon inhibition of USP10, and impairs the migratory capability of HCC cells [51]. In osteosarcoma (OS), USP10 stabilizes GSK3β and promotes transcription of *ULK1*. Spautin-1 delays tumor progression by inhibiting the USP10-GSK3β-ULK1 axis, leading to a decrease in *ULK1* transcription, thus inhibits autophagy and OS cell proliferation and invasion. In in vivo experiments, spautin-1 combined with cisplatin significantly reduces tumor progression [40], suggesting that spautin-1 may be usable for clinical therapy of OS.

### 5.2. Wu-5

Wu-5 binds directly to USP10 and inhibits its deubiquitination activity. Wu-5 was observed to induce an augmentation in the ubiquitination of the FLT3-ITD and facilitate the proteasome-mediated degradation of FLT3-ITD. Overexpression of USP10 completely reversed the effect of Wu-5 on FLT3-ITD degradation and cell death. Wu-5 shows a potential application for overcoming drug resistance in AML therapy, especially for FLT3-ITD-positive subtypes, which are often accompanied by poor prognosis and risk of conventional treatment failure [168]. However, the role of Wu-5 in inhibition of other types of cancer has not been determined.

## 6. Conclusions

USP10 has emerged as a key protein in regulation of tumorigenesis and progression. USP10 has either tumor suppressing or oncogenic function dependent on the cellular function of its substrates and the associated downstream signaling context. Most of current studies have found that USP10 play oncogenic roles in cancers, particularly in the cancers containing the TP53 mutants, SMAD4 or FLT3-ITD. Thus, USP10 is a valid target for cancer therapy.

Although targeting USP10 has great potential for cancer therapy, current approaches utilizing USP10 as a cancer therapeutic target are still far from clinical application. Because USP10 has dual roles in cancers, the application of the USP10 inhibitors as anti-cancer drugs should take a cautious approach. It is necessary to determine the expression of tumor suppressor substrates of USP10, such as wild-type TP53 or PTEN, before using USP10 inhibitor-based cancer therapy. At the same time, it should be closely monitored whether the USP10 inhibitor induces neoplasms in normal tissues. Apparently, more preclinical research is needed to develop the USP10 inhibitors as anti-cancer drugs for cancer therapy in future.

## Figures and Tables

**Table 2:** Cellular signaling pathways regulated by USP10.

	Signaling Pathway	Substrate Protein	USP10 Roles	Reference
Anti-cancer	TP53 transcription	TP53	Stabilizing TP53.	[92]
	PI3K/AKT/mTOR	PTEN	Stabilizes PTEN and inhibits AKT phosphorylation and mTOR activation.	[119]
	AMPK	AMPKα	Stabilization of AMPKα inhibits mTOR activation	[119]
	Wnt/β-catenin	AXIN1	Stabilizes AXIN1 and inhibits β-catenin signaling.	[88]
Pro-cancer	MAPK	TP53 mutant	Mutant TP53 activates the MAPK signaling pathway, promoting cell survival and clonal expansion, thereby driving tumor metastasis.	[120]
	Notch	NICD	Stabilizes NICD, antagonizes E3 ligase-mediated ubiquitination, and enhances Notch signaling pathway duration.	[70]
	Hippo	YAP/TAZ	USP10 promotes cancer cell proliferation by directly interacting with YAP/TAZ to stabilize the YAP/TAZ proteins.	[83]
	ERα	ERα	USP10 prevents ERα degradation by the ubiquitin-proteasome system. It enhances its transcriptional activity, promotes expression of downstream target genes, and drives tumor growth.	[59]
	Nrf2	Sirt6	Blocking Sirt6 degradation by the proteasome and activating pathways to attenuate oxidative stress and apoptosis.	[23]
	Bcr-Abl	SKP2	USP10 amplifies Bcr-Abl activation by deubiquitinating and stabilizing SKP2.	[77]
	TGF-β/SMAD	SMAD4	Deubiquitination of SMAD4, activation of TGF-β signaling, and promotion of hepatocellular carcinoma metastasis.	[51]
	RAF/MEK/ERK	RAF1	USP10 overexpression increases RAF1 activation of the downstream MEK/ERK signaling pathway, promotes cell proliferation and migration, and inhibits apoptosis.	[74]
	AR	G3BP2	Stabilizes G3BP2, enhances the transcriptional activity of the AR signaling pathway, and creates a pro-cancer cycle.	[26]
	EGFR	INPP4B	Knockdown of USP10 leads to elevated INPP4B protein levels and directly reduces EGFR-mediated activation of the PI3K/AKT signaling pathway.	[121]

**Table 3:** Cancers regulated by USP10.

Role 10.	Cancer	Mechanism	Reference
Inhibition	Lung Cancer	USP10 inhibits the proliferation of non-small cell lung cancer by directly binding to PTEN and suppressing the AKT signaling pathway.	[94]
	Liver Cancer	USP10 stabilizes PTEN and AMPKα proteins to form the USP10-PTEN-AMPKα axis, thereby blocking the mTOR signaling pathway and inhibiting hepatocellular carcinoma cell proliferation.	[119]
	Colorectal Cancer	USP10 binds AXIN1 to β-catenin and inhibits β-catenin by promoting phase separation, significantly suppressing colorectal cancer cell growth.	[88]
	Gastric Cancer	Molecular mechanisms indicate that USP10 –stabilizes TNFRSF10B via deubiquitination, thereby inhibiting the epithelial–mesenchymal transition (EMT).	[95]
	Renal Cell Carcinoma	USP10 deficiency promotes the ubiquitination and degradation of Sirt6 and TP53, thereby activating c-Myc and accelerating tumor progression. Overexpression of USP10 suppresses c-Myc transcriptional activity, blocks cell cycle progression, and inhibits renal tumor growth.	[92,150]
	Thyroid Cancer	USP10 expression is downregulated in thyroid cancer cells. Restoring USP10 expression inhibits cell invasion, migration, and epithelial–mesenchymal transition (EMT) while promoting apoptosis. This effect is associated with inhibition of the PI3K/AKT pathway.	[151]
Promotion	Prostate Cancer	USP10 deubiquitinates G3BP2, thereby inhibiting TP53 nuclear transport and promoting prostate cancer cell proliferation.	[26]
	Breast Cancer	USP10 deubiquitinates and stabilizes CD44, a key marker in breast cancer cells, thereby activating the PDGFRβ/STAT3 signaling pathway and promoting breast cancer progression.	[57]
	Pancreatic Ductal Adenocarcinoma	The tumor suppressor gene N1DARP promotes Notch1 degradation by disrupting its interaction with USP10, thereby inhibiting pancreatic cancer progression.	[71]
	Lung cancer	USP10 deubiquitinates and stabilizes the oncogene EIF4G1, thereby promoting proliferation, migration, and invasion in non-small cell lung cancer cells.	[58]
	Liver cancer	USP10 stabilizes SMAD4 protein levels by cleaving ubiquitin chains, thereby activating the TGF-β/SMAD4 signaling pathway and ultimately promoting hepatocellular carcinoma metastasis.	[51]
	Leukemia	USP10 deubiquitinates and stabilizes FLT3-ITD mutant proteins while also deubiquitinating and stabilizing SYK, a key FLT3 regulator, thereby promoting AML cell proliferation.	[25]

## Data Availability

No new data were created or analyzed in this study.

## References

[1] Bhattacharya U., Neizer-Ashun F., Mukherjee P., Bhattacharya R. (2020). When the Chains Do Not Break: The Role of USP10 in Physiology and Pathology. Cell Death Dis..

[2] Jee S.-C., Cheong H. (2023). Autophagy/Mitophagy Regulated by Ubiquitination: A Promising Pathway in Cancer Therapeutics. Cancers.

[3] Doleschal M.N., Miller J., Jain S., Zakharov A.V., Rai G., Simeonov A., Baljinnyam B., Zhuang Z. (2024). Cell-Based Covalent-Capture Deubiquitinase Assay for Inhibitor Discovery. ACS Pharmacol. Transl. Sci..

[4] Cruz L., Soares P., Correia M. (2021). Ubiquitin-Specific Proteases: Players in Cancer Cellular Processes. Pharmaceuticals.

[5] Mevissen T.E.T., Komander D. (2017). Mechanisms of Deubiquitinase Specificity and Regulation. Annu. Rev. Biochem..

[6] Cho J., Park J., Kim E.E., Song E.J. (2020). Assay Systems for Profiling Deubiquitinating Activity. Int. J. Mol. Sci..

[7] Zeng C., Zhao C., Ge F., Li Y., Cao J., Ying M., Lu J., He Q., Yang B., Dai X. (2020). Machado-Joseph Deubiquitinases: From Cellular Functions to Potential Therapy Targets. Front. Pharmacol..

[8] Komander D., Clague M.J., Urbé S. (2009). Breaking the Chains: Structure and Function of the Deubiquitinases. Nat. Rev. Mol. Cell Biol..

[9] Miranda R., Anson F., Smith S.T., Ultsch M., Tenorio C.A., Rougé L., Farrell B., Adaligil E., Holden J.K., Harris S.F. (2025). Discovery and Characterization of Potent Macrocycle Inhibitors of Ubiquitin-Specific Protease-7. Structure.

[10] Keijzer N., Priyanka A., Stijf-Bultsma Y., Fish A., Gersch M., Sixma T.K. (2024). Variety in the USP Deubiquitinase Catalytic Mechanism. Life Sci. Alliance.

[11] Ronau J.A., Beckmann J.F., Hochstrasser M. (2016). Substrate Specificity of the Ubiquitin and Ubl Proteases. Cell Res..

[12] Yuan T., Yan F., Ying M., Cao J., He Q., Zhu H., Yang B. (2018). Inhibition of Ubiquitin-Specific Proteases as a Novel Anticancer Therapeutic Strategy. Front. Pharmacol..

[13] Gao H., Xi Z., Dai J., Xue J., Guan X., Zhao L., Chen Z., Xing F. (2024). Drug Resistance Mechanisms and Treatment Strategies Mediated by Ubiquitin-Specific Proteases (USPs) in Cancers: New Directions and Therapeutic Options. Mol. Cancer.

[14] Gao H., Yin J., Ji C., Yu X., Xue J., Guan X., Zhang S., Liu X., Xing F. (2023). Targeting Ubiquitin Specific Proteases (USPs) in Cancer Immunotherapy: From Basic Research to Preclinical Application. J. Exp. Clin. Cancer Res..

[15] Nijman S.M.B., Luna-Vargas M.P.A., Velds A., Brummelkamp T.R., Dirac A.M.G., Sixma T.K., Bernards R. (2005). A Genomic and Functional Inventory of Deubiquitinating Enzymes. Cell.

[16] Ye Z., Chen J., Huang P., Xuan Z., Zheng S. (2022). Ubiquitin-Specific Peptidase 10, a Deubiquitinating Enzyme: Assessing Its Role in Tumor Prognosis and Immune Response. Front. Oncol..

[17] Yang J.-R., Lu Y.-B., Su H.-X., Xiao Y., Pan Q., Su F., Zhang X.-B., Zhu K.-L., Guan Q.-L., Ling X.-L. (2023). USP10 Promotes the Progression of Triple-Negative Breast Cancer by Enhancing the Stability of TCF4 Protein. Biochem. Pharmacol..

[18] Ma C., Lin Z., Yao J., Qin W., Wang X., Li Q., Ye Y., Liu X., Chen F., Hu J. (2024). Loss of USP10 Promotes Hepatocellular Carcinoma Proliferation by Regulating the Serine Synthesis Pathway through Inhibition of LKB1 Activity. Cancer Sci..

[19] Chen X., Ma Y., Liu H., Wang Y. (2025). Multifunctional Regulation and Treatment of Ubiquitin Specific Protease 10. Biochem. Pharmacol..

[20] Shen C., Li J., Zhang Q., Tao Y., Li R., Ma Z., Wang Z. (2022). LncRNA GASAL1 Promotes Hepatocellular Carcinoma Progression by Up-Regulating USP10-Stabilized PCNA. Exp. Cell Res..

[21] Hu C., Zhang M., Moses N., Hu C.-L., Polin L., Chen W., Jang H., Heyza J., Malysa A., Caruso J.A. (2020). The USP10-HDAC6 Axis Confers Cisplatin Resistance in Non-Small Cell Lung Cancer Lacking Wild-Type P53. Cell Death Dis..

[22] Wang Y., Chang F., Li Z., Duan C., Sun X., Wang S., Wei D., Li W., Qian Y., Cao S. (2025). CircTP53/USP10/P53 Signaling Axis as a Novel Regulator of Progression and Prognosis of Head and Neck Squamous Cell Carcinoma. Adv. Sci..

[23] Gao F., Qian M., Liu G., Ao W., Dai D., Yin C. (2021). USP10 Alleviates Sepsis-Induced Acute Kidney Injury by Regulating Sirt6-Mediated Nrf2/ARE Signaling Pathway. J. Inflamm..

[24] Bhattacharya U., Thavathiru E., Neizer-Ashun F., Xu C., Gatalica Z., Dwivedi S.K.D., Dey A., Mukherjee P., Bhattacharya R. (2022). The Deubiquitinase USP10 Protects Pancreatic Cancer Cells from Endoplasmic Reticulum Stress. NPJ Precis. Oncol..

[25] Tao L., Liu X., Jiang X., Zhang K., Wang Y., Li X., Jiang S., Han T. (2022). USP10 as a Potential Therapeutic Target in Human Cancers. Genes.

[26] Takayama K.-I., Suzuki T., Fujimura T., Takahashi S., Inoue S. (2018). Association of USP10 with G3BP2 Inhibits P53 Signaling and Contributes to Poor Outcome in Prostate Cancer. Mol. Cancer Res..

[27] Wang C., Ni J., Zhai D., Xu Y., Wu Z., Chen Y., Liu N., Du J., Shen Y., Liu G. (2024). Stress-Induced Epinephrine Promotes Hepatocellular Carcinoma Progression via the USP10-PLAGL2 Signaling Loop. Exp. Mol. Med..

[28] Zhao Q., Zhang R., Wang Y., Li T., Xue J., Chen Z. (2024). FOXQ1, Deubiquitinated by USP10, Alleviates Sepsis-Induced Acute Kidney Injury by Targeting the CREB5/NF-κB Signaling Axis. Biochim. Biophys. Acta Mol. Basis Dis..

[29] Liu H., Ding J., Köhnlein K., Urban N., Ori A., Villavicencio-Lorini P., Walentek P., Klotz L.-O., Hollemann T., Pfirrmann T. (2020). The GID Ubiquitin Ligase Complex Is a Regulator of AMPK Activity and Organismal Lifespan. Autophagy.

[30] Zhang D.-H., Zhang J.-L., Huang Z., Wu L.-M., Wang Z.-M., Li Y.-P., Tian X.-Y., Kong L.-Y., Yao R., Zhang Y.-Z. (2020). Deubiquitinase Ubiquitin-Specific Protease 10 Deficiency Regulates Sirt6 Signaling and Exacerbates Cardiac Hypertrophy. J. Am. Heart Assoc..

[31] Mao S., Yu N., Wang W., Mao Y., Du Y., Zhao Q., Gu X., Kang J. (2025). Ubiquitin-Specific Peptidase 10 Attenuates Bleomycin-Induced Pulmonary Fibrosis via Modulating Autophagy Depending on Sirtuin 6-Mediated AKT/mTOR. Cell Biol. Toxicol..

[32] Lian F., Dong D., Pu J., Yang G., Yang J., Yang S., Wang Y., Zhao B., Lu M. (2024). Ubiquitin-Specific Peptidase 10 Attenuates the Ferroptosis to Promote Thyroid Cancer Malignancy by Facilitating GPX4 via Elevating SIRT6. Environ. Toxicol..

[33] Wang X., Fan X., Zhang J., Wang F., Chen J., Wen Y., Wang L., Li T., Li H., Gu H. (2024). hnRNPA2B1 Represses the Disassembly of Arsenite-Induced Stress Granules and Is Essential for Male Fertility. Cell Rep..

[34] Piatnitskaia S., Takahashi M., Kitaura H., Katsuragi Y., Kakihana T., Zhang L., Kakita A., Iwakura Y., Nawa H., Miura T. (2019). USP10 Is a Critical Factor for Tau-Positive Stress Granule Formation in Neuronal Cells. Sci. Rep..

[35] Sheinberger J., Shav-Tal Y. (2017). mRNPs Meet Stress Granules. FEBS Lett..

[36] Tolay N., Buchberger A. (2022). Role of the Ubiquitin System in Stress Granule Metabolism. Int. J. Mol. Sci..

[37] Song D., Kuang L., Yang L., Wang L., Li H., Li X., Zhu Z., Shi C., Zhu H., Gong W. (2022). Yin and Yang Regulation of Stress Granules by Caprin-1. Proc. Natl. Acad. Sci. USA.

[38] Schulte T., Panas M.D., Han X., Williams L., Kedersha N., Fleck J.S., Tan T.J.C., Dopico X.C., Olsson A., Morro A.M. (2023). Caprin-1 Binding to the Critical Stress Granule Protein G3BP1 Is Influenced by pH. Open Biol..

[39] Meyer C., Garzia A., Morozov P., Molina H., Tuschl T. (2020). The G3BP1-Family-USP10 Deubiquitinase Complex Rescues Ubiquitinated 40S Subunits of Ribosomes Stalled in Translation from Lysosomal Degradation. Mol. Cell.

[40] Feng Z., Ou Y., Deng X., Deng M., Yan X., Chen L., Zhou F., Hao L. (2024). Deubiquitinase USP10 Promotes Osteosarcoma Autophagy and Progression through Regulating GSK3β-ULK1 Axis. Cell Biosci..

[41] Xin S.-L., Yu Y.-Y. (2022). Ubiquitin-Specific Peptidase 10 Ameliorates Hepatic Steatosis in Nonalcoholic Steatohepatitis Model by Restoring Autophagic Activity. Dig. Liver Dis..

[42] Jia R., Bonifacino J.S. (2021). The Ubiquitin Isopeptidase USP10 Deubiquitinates LC3B to Increase LC3B Levels and Autophagic Activity. J. Biol. Chem..

[43] Liu J., Xia H., Kim M., Xu L., Li Y., Zhang L., Cai Y., Norberg H.V., Zhang T., Furuya T. (2011). Beclin1 Controls the Levels of P53 by Regulating the Deubiquitination Activity of USP10 and USP13. Cell.

[44] Kubaichuk K., Kietzmann T. (2023). USP10 Contributes to Colon Carcinogenesis via mTOR/S6K Mediated HIF-1α but Not HIF-2α Protein Synthesis. Cells.

[45] Yang R., Chen H., Xing L., Wang B., Hu M., Ou X., Chen H., Deng Y., Liu D., Jiang R. (2022). Hypoxia-Induced circWSB1 Promotes Breast Cancer Progression through Destabilizing P53 by Interacting with USP10. Mol. Cancer.

[46] Fu M., Li J., Xuan Z., Zheng Z., Liu Y., Zhang Z., Zheng J., Zhong M., Liu B., Du Y. (2024). NDR1 Mediates PD-L1 Deubiquitination to Promote Prostate Cancer Immune Escape via USP10. Cell Commun. Signal.

[47] Shen G.-Y., Zhang Y., Huang R.-Z., Huang Z.-Y., Yang L.-Y., Chen D.-Z., Yang S.-B. (2024). FOXP4-AS1 Promotes CD8+ T Cell Exhaustion and Esophageal Cancer Immune Escape through USP10-Stabilized PD-L1. Immunol. Res..

[48] Liu X., Chen B., Chen J., Su Z., Sun S. (2022). Deubiquitinase Ubiquitin-Specific Peptidase 10 Maintains Cysteine Rich Angiogenic Inducer 61 Expression via Yes1 Associated Transcriptional Regulator to Augment Immune Escape and Metastasis of Pancreatic Adenocarcinoma. Cancer Sci..

[49] Li B., Qi Z.-P., He D.-L., Chen Z.-H., Liu J.-Y., Wong M.-W., Zhang J.-W., Xu E.-P., Shi Q., Cai S.-L. (2021). NLRP7 Deubiquitination by USP10 Promotes Tumor Progression and Tumor-Associated Macrophage Polarization in Colorectal Cancer. J. Exp. Clin. Cancer Res..

[50] Gao D., Zhang Z., Xu R., He Z., Li F., Hu Y., Chen H., Lu J., Cao X., Liu Y. (2022). The Prognostic Value and Immune Infiltration of USP10 in Pan-Cancer: A Potential Therapeutic Target. Front. Oncol..

[51] Yuan T., Chen Z., Yan F., Qian M., Luo H., Ye S., Cao J., Ying M., Dai X., Gai R. (2020). Deubiquitinating Enzyme USP10 Promotes Hepatocellular Carcinoma Metastasis through Deubiquitinating and Stabilizing Smad4 Protein. Mol. Oncol..

[52] Ren J., Yu P., Liu S., Li R., Niu X., Chen Y., Zhang Z., Zhou F., Zhang L. (2023). Deubiquitylating Enzymes in Cancer and Immunity. Adv. Sci..

[53] Cao Y.-F., Xie L., Tong B.-B., Chu M.-Y., Shi W.-Q., Li X., He J.-Z., Wang S.-H., Wu Z.-Y., Deng D.-X. (2023). Targeting USP10 Induces Degradation of Oncogenic ANLN in Esophageal Squamous Cell Carcinoma. Cell Death Differ..

[54] Fang X.-L., Li Q.-J., Lin J.-Y., Huang C.-L., Huang S.-Y., Tan X.-R., He S.-W., Zhu X.-H., Li J.-Y., Gong S. (2024). Transcription Factor ATMIN Facilitates Chemoresistance in Nasopharyngeal Carcinoma. Cell Death Dis..

[55] Shi Y., Ding J., Ling X., Xu D., Shen Y., Qin X. (2025). USP10 Stabilizes BAZ1A to Drive Tumor Stemness via an Epigenetic Mechanism in Head and Neck Squamous Cell Carcinoma. Cell Death Dis..

[56] Sun T., Xu Y.-J., Jiang S.-Y., Xu Z., Cao B.-Y., Sethi G., Zeng Y.-Y., Kong Y., Mao X.-L. (2021). Suppression of the USP10/CCND1 Axis Induces Glioblastoma Cell Apoptosis. Acta Pharmacol. Sin..

[57] Sethi A., Mishra S., Upadhyay V., Dubey P., Siddiqui S., Singh A.K., Chowdhury S., Srivastava S., Srivastava P., Sahoo P. (2024). USP10 Deubiquitinates and Stabilizes CD44 Leading to Enhanced Breast Cancer Cell Proliferation, Stemness and Metastasis. Biochem. J..

[58] Li F., He Z., Zhang X., Gao D., Xu R., Zhang Z., Cao X., Shan Q., Liu Y., Xu Z. (2024). USP10 Promotes Cell Proliferation, Migration, and Invasion in NSCLC through Deubiquitination and Stabilization of EIF4G1. Sci. Rep..

[59] Li H., Liu Y., Cai Z., Li K., Gao S., Lan A., Shu D., He K., Liu X., Peng Y. (2025). ARL3 Enhances ERα Stability via USP10 Deubiquitination to Promote Endocrine Resistance and Drive Mitochondrial Metabolic Reprogramming in HR+ Breast Cancer. Adv. Sci..

[60] Tong W., Xie X., Shu Z., Nie J., Yang X., Yang F., Liu Z., Liu J. (2025). SPICE1 Promotes Osteosarcoma Growth by Enhancing the Deubiquitination of FASN Mediated by USP10. J. Transl. Med..

[61] Weisberg E.L., Schauer N.J., Yang J., Lamberto I., Doherty L., Bhatt S., Nonami A., Meng C., Letai A., Wright R. (2017). Inhibition of USP10 Induces Degradation of Oncogenic FLT3. Nat. Chem. Biol..

[62] Wang J., Gan L., Liu F., Yang Q., Deng Q., Jiang D., Zhang C., Zhang L., Wang X. (2024). USP10 Promotes Pancreatic Ductal Adenocarcinoma Progression by Attenuating FOXC1 Protein Degradation to Activate the WNT Signaling Pathway. Int. J. Biol. Sci..

[63] Li Q.-J., Fang X.-L., Li Y.-Q., Lin J.-Y., Huang C.-L., He S.-W., Huang S.-Y., Li J.-Y., Gong S., Liu N. (2024). DCAF7 Acts as A Scaffold to Recruit USP10 for G3BP1 Deubiquitylation and Facilitates Chemoresistance and Metastasis in Nasopharyngeal Carcinoma. Adv. Sci..

[64] Chen Y., Shen H., Wang Z., Huang C., Zhang H., Shao Y., Tong Y., Xu L., Lu Y., Fu Z. (2024). Recruitment of USP10 by GCS1 to Deubiquitinate GRP78 Promotes the Progression of Colorectal Cancer via Alleviating Endoplasmic Reticulum Stress. J. Exp. Clin. Cancer Res..

[65] Zhao W., Lu D., Liu L., Cai J., Zhou Y., Yang Y., Zhang Y., Zhang J. (2017). Insulin-like Growth Factor 2 mRNA Binding Protein 3 (IGF2BP3) Promotes Lung Tumorigenesis via Attenuating P53 Stability. Oncotarget.

[66] Shi J., Zhang Q., Yin X., Ye J., Gao S., Chen C., Yang Y., Wu B., Fu Y., Zhang H. (2023). Stabilization of IGF2BP1 by USP10 Promotes Breast Cancer Metastasis via CPT1A in an m6A-Dependent Manner. Int. J. Biol. Sci..

[67] Wang H., Cui C., Li W., Wu H., Sha J., Pan J., Xue W. (2025). AGD1/USP10/METTL13 Complexes Enhance Cancer Stem Cells Proliferation and Diminish the Therapeutic Effect of Docetaxel via CD44 m6A Modification in Castration Resistant Prostate Cancer. J. Exp. Clin. Cancer Res..

[68] Li P., Yang L., Park S.Y., Liu F., Li A.H., Zhu Y., Sui H., Gao F., Li L., Ye L. (2024). Stabilization of MOF (KAT8) by USP10 Promotes Esophageal Squamous Cell Carcinoma Proliferation and Metastasis through Epigenetic Activation of ANXA2/Wnt Signaling. Oncogene.

[69] Ouyang S.-W., Liu T.-T., Liu X.-S., Zhu F.-X., Zhu F.-M., Liu X.-N., Peng Z.-H. (2019). USP10 Regulates Musashi-2 Stability via Deubiquitination and Promotes Tumour Proliferation in Colon Cancer. FEBS Lett..

[70] Lim R., Sugino T., Nolte H., Andrade J., Zimmermann B., Shi C., Doddaballapur A., Ong Y.T., Wilhelm K., Fasse J.W.D. (2019). Deubiquitinase USP10 Regulates Notch Signaling in the Endothelium. Science.

[71] Zhai S., Lin J., Ji Y., Zhang R., Zhang Z., Cao Y., Liu Y., Tang X., Liu J., Liu P. (2023). A Microprotein N1DARP Encoded by LINC00261 Promotes Notch1 Intracellular Domain (N1ICD) Degradation via Disrupting USP10-N1ICD Interaction to Inhibit Chemoresistance in Notch1-Hyperactivated Pancreatic Cancer. Cell Discov..

[72] Li T.-J., Jin K.-Z., Zhou H.-Y., Liao Z.-Y., Zhang H.-R., Shi S.-M., Lin M.-X., Chai S.-J., Fei Q.-L., Ye L.-Y. (2023). Deubiquitinating PABPC1 by USP10 Upregulates CLK2 Translation to Promote Tumor Progression in Pancreatic Ductal Adenocarcinoma. Cancer Lett..

[73] Du X., Yu R., Yan C., Dong P., Wei C., Wang B., Zhang C., He Y., Wei Y., Han L. (2025). USP10 Promotes the Progression and Attenuates Gemcitabine Chemotherapy Sensitivity via Stabilizing PLK1 in PDAC. Cell Death Dis..

[74] Chen Q., Hang Y., Zhang T., Tan L., Li S., Jin Y. (2018). USP10 Promotes Proliferation and Migration and Inhibits Apoptosis of Endometrial Stromal Cells in Endometriosis through Activating the Raf-1/MEK/ERK Pathway. Am. J. Physiol. Cell Physiol..

[75] Li H., Chai L., Ding Z., He H. (2022). CircCOL1A2 Sponges MiR-1286 to Promote Cell Invasion and Migration of Gastric Cancer by Elevating Expression of USP10 to Downregulate RFC2 Ubiquitination Level. J. Microbiol. Biotechnol..

[76] Qiu W., Xiao Z., Yang Y., Jiang L., Song S., Qi X., Chen Y., Yang H., Liu J., Chu L. (2023). USP10 Deubiquitinates RUNX1 and Promotes Proneural-to-Mesenchymal Transition in Glioblastoma. Cell Death Dis..

[77] Liao Y., Liu N., Xia X., Guo Z., Li Y., Jiang L., Zhou R., Tang D., Huang H., Liu J. (2019). USP10 Modulates the SKP2/Bcr-Abl Axis via Stabilizing SKP2 in Chronic Myeloid Leukemia. Cell Discov..

[78] Zhu W., Ye B., Yang S., Li Y. (2023). USP10 Promotes Intrahepatic Cholangiocarcinoma Cell Survival and Stemness via SNAI1 Deubiquitination. J. Mol. Histol..

[79] Wang H., Liang L., Xie Y., Gong H., Fan F., Wen C., Jiang Y., Lei S., Qiu X., Peng H. (2025). Pseudokinase TRIB3 Stabilizes SSRP1 via USP10-Mediated Deubiquitination to Promote Multiple Myeloma Progression. Oncogene.

[80] Yang J., Meng C., Weisberg E., Case A., Lamberto I., Magin R.S., Adamia S., Wang J., Gray N., Liu S. (2020). Inhibition of the Deubiquitinase USP10 Induces Degradation of SYK. Br. J. Cancer.

[81] Deng C.-C., Zhu D.-H., Chen Y.-J., Huang T.-Y., Peng Y., Liu S.-Y., Lu P., Xue Y.-H., Xu Y.-P., Yang B. (2019). TRAF4 Promotes Fibroblast Proliferation in Keloids by Destabilizing P53 via Interacting with the Deubiquitinase USP10. J. Investig. Dermatol..

[82] Liu X., Zhang S., An Y., Xu B., Yan G., Sun M. (2025). USP10/XAB2/ANXA2 Axis Promotes DNA Damage Repair to Enhance Chemoresistance to Oxaliplatin in Colorectal Cancer. J. Exp. Clin. Cancer Res..

[83] Zhu H., Yan F., Yuan T., Qian M., Zhou T., Dai X., Cao J., Ying M., Dong X., He Q. (2020). USP10 Promotes Proliferation of Hepatocellular Carcinoma by Deubiquitinating and Stabilizing YAP/TAZ. Cancer Res..

[84] Lin C., Lin P., Yao H., Liu S., Lin X., He R., Teng Z., Zuo X., Li Y., Ye J. (2024). Modulation of YBX1-Mediated PANoptosis Inhibition by PPM1B and USP10 Confers Chemoresistance to Oxaliplatin in Gastric Cancer. Cancer Lett..

[85] Gao Y., Zhang X., Xiao L., Zhai C., Yi T., Wang G., Wang E., Ji X., Hu L., Shen G. (2019). Usp10 Modulates the Hippo Pathway by Deubiquitinating and Stabilizing the Transcriptional Coactivator Yorkie. Int. J. Mol. Sci..

[86] Huang Q., Zhang R., Xia Y., Shen J., Dong H., Li X., Tao D., Xie D., Liu L. (2023). DAB2IP Suppresses Invadopodia Formation through Destabilizing ALK by Interacting with USP10 in Breast Cancer. iScience.

[87] Deng M., Yang X., Qin B., Liu T., Zhang H., Guo W., Lee S.B., Kim J.J., Yuan J., Pei H. (2016). Deubiquitination and Activation of AMPK by USP10. Mol. Cell.

[88] Wang Y., Mao A., Liu J., Li P., Zheng S., Tong T., Li Z., Zhang H., Ma L., Lin J. (2023). USP10 Strikes down β-Catenin by Dual-Wielding Deubiquitinase Activity and Phase Separation Potential. Cell Chem. Biol..

[89] Xu Y.-J., Zeng K., Ren Y., Mao C.-Y., Ye Y.-H., Zhu X.-T., Sun Z.-Y., Cao B.-Y., Zhang Z.-B., Xu G.-Q. (2023). Inhibition of USP10 Induces Myeloma Cell Apoptosis by Promoting Cyclin D3 Degradation. Acta Pharmacol. Sin..

[90] Chen Q., Xiong X., Sun Y. (2024). USP10 Deubiquitylates and Stabilizes DIRAS2 to Suppress the Growth of Pancreatic Cancer Cells. MedComm.

[91] Zeng Z., Li D., Yu T., Huang Y., Yan H., Gu L., Yuan J. (2019). Association and Clinical Implication of the USP10 and MSH2 Proteins in Non-Small Cell Lung Cancer. Oncol. Lett..

[92] Yuan J., Luo K., Zhang L., Cheville J.C., Lou Z. (2010). USP10 Regulates P53 Localization and Stability by Deubiquitinating P53. Cell.

[93] Zhang X.M., Gavande N., Parajuli P., Bepler G. (2021). Implications of the USP10-HDAC6 Axis in Lung Cancer–A Path to Precision Medicine. J. Cancer Biol..

[94] He Y., Jiang S., Mao C., Zheng H., Cao B., Zhang Z., Zhao J., Zeng Y., Mao X. (2021). The Deubiquitinase USP10 Restores PTEN Activity and Inhibits Non-Small Cell Lung Cancer Cell Proliferation. J. Biol. Chem..

[95] Zeng Z., Li Y., Zhou H., Li M., Ye J., Li D., Zhu Y., Zhang Y., Zhang X., Deng Y. (2024). System-Wide Identification of Novel de-Ubiquitination Targets for USP10 in Gastric Cancer Metastasis through Multi-Omics Screening. BMC Cancer.

[96] Luo D., Tang H., Tan L., Zhang L., Wang L., Cheng Q., Lei X., Wu J. (2024). lncRNA JPX Promotes Tumor Progression by Interacting with and Destabilizing YTHDF2 in Cutaneous Melanoma. Mol. Cancer Res..

[97] Sun L., Yu J., Guinney J., Qin B., Sinicrope F.A. (2023). USP10 Regulates ZEB1 Ubiquitination and Protein Stability to Inhibit ZEB1-Mediated Colorectal Cancer Metastasis. Mol. Cancer Res..

[98] Luo Y., Zhang X., Chen R., Li R., Liu Y., Zhang J., Liu Q., Si M., Liu J., Wu B. (2022). USP10 Regulates B Cell Response to SARS-CoV-2 or HIV-1 Nanoparticle Vaccines through Deubiquitinating AID. Signal Transduct. Target. Ther..

[99] Xia X., Hu T., He J., Xu Q., Yu C., Liu X., Shao Z., Liao Y., Huang H., Liu N. (2020). USP10 Deletion Inhibits Macrophage-Derived Foam Cell Formation and Cellular-Oxidized Low Density Lipoprotein Uptake by Promoting the Degradation of CD36. Aging.

[100] Tang X., Weng R., Guo G., Wei J., Wu X., Chen B., Liu S., Zhong Z., Chen X. (2023). USP10 Regulates Macrophage Inflammation Responses via Stabilizing NEMO in LPS-Induced Sepsis. Inflamm. Res..

[101] Zeng L., Zhu Y., Cui X., Chi J., Uddin A., Zhou Z., Song X., Dai M., Cristofanilli M., Kalinsky K. (2024). Tuning Immune-Cold Tumor by Suppressing USP10/B7-H4 Proteolytic Axis Reinvigorates Therapeutic Efficacy of ADCs. Adv. Sci..

[102] Chen W., Xu L., Deng H., Zhu Z., Yang D. (2025). Role of USP10/METTL3/CXCR4 Axis in Immunotherapy of Castration-Resistant Prostate Cancer. Iran. J. Allergy Asthma Immunol..

[103] Chen J., Qi D., Hu H., Wang X., Lin W. (2024). Unconventional Posttranslational Modification in Innate Immunity. Cell Mol. Life Sci..

[104] Luo P., Qin C., Zhu L., Fang C., Zhang Y., Zhang H., Pei F., Tian S., Zhu X.-Y., Gong J. (2018). Ubiquitin-Specific Peptidase 10 (USP10) Inhibits Hepatic Steatosis, Insulin Resistance, and Inflammation Through Sirt6. Hepatology.

[105] Pan L., Chen Z., Wang L., Chen C., Li D., Wan H., Li B., Shi G. (2014). Deubiquitination and Stabilization of T-Bet by USP10. Biochem. Biophys. Res. Commun..

[106] Zhou J., Wang T., Chen Z., Zhang L., Zou J., Ma X., Qiu T. (2020). Ubiquitin-Specific Peptidase 10 Protects Against Hepatic Ischaemic/Reperfusion Injury via TAK1 Signalling. Front. Immunol..

[107] Wang W., Huang X., Xin H.-B., Fu M., Xue A., Wu Z.-H. (2015). TRAF Family Member-Associated NF-κB Activator (TANK) Inhibits Genotoxic Nuclear Factor κB Activation by Facilitating Deubiquitinase USP10-Dependent Deubiquitination of TRAF6 Ligase. J. Biol. Chem..

[108] Wang B., Hu L., Zhang Z., Yin Y., Zheng L., Wu H., Cheng Y., Zhang G. (2025). UBE2S-Mediated Deubiquitination of GLUT1 via USP10 Regulates Glucose Metabolic Reprogramming and Immune Microenvironment to Promote Fibrosis in Endometriosis. J. Transl. Med..

[109] Takahashi M., Higuchi M., Makokha G.N., Matsuki H., Yoshita M., Tanaka Y., Fujii M. (2013). HTLV-1 Tax Oncoprotein Stimulates ROS Production and Apoptosis in T Cells by Interacting with USP10. Blood.

[110] Huang Y., Zhang J., Zhu Y., Zhao R., Xie Z., Qu X., Duan Y., Li N., Tang D., Luo X. (2026). BMP9 Alleviates Iron Accumulation-Induced Osteoporosis via the USP10/FOXO1/GPX4 Axis. J. Adv. Res..

[111] Takahashi M., Higuchi M., Matsuki H., Yoshita M., Ohsawa T., Oie M., Fujii M. (2013). Stress Granules Inhibit Apoptosis by Reducing Reactive Oxygen Species Production. Mol. Cell Biol..

[112] Lin P., Lin C., Teng Z., Liu S., Lin X., He R., Yao H., Ye J., Zhu G. (2025). USP10-Mediated Ku70/80 Stabilization Inhibits PANoptosis and Promotes Chemoresistance in Colorectal Cancer. Oncogene.

[113] Sango J., Kakihana T., Takahashi M., Katsuragi Y., Anisimov S., Komatsu M., Fujii M. (2022). USP10 Inhibits the Dopamine-Induced Reactive Oxygen Species-Dependent Apoptosis of Neuronal Cells by Stimulating the Antioxidant Nrf2 Activity. J. Biol. Chem..

[114] Takahashi M., Kitaura H., Kakita A., Kakihana T., Katsuragi Y., Nameta M., Zhang L., Iwakura Y., Nawa H., Higuchi M. (2018). USP10 Is a Driver of Ubiquitinated Protein Aggregation and Aggresome Formation to Inhibit Apoptosis. iScience.

[115] Pietrzak J., Spickett C.M., Płoszaj T., Virág L., Robaszkiewicz A. (2018). PARP1 Promoter Links Cell Cycle Progression with Adaptation to Oxidative Environment. Redox Biol..

[116] Zhang D., Wang X., Lu S., Gao Y., Zhu G., Li G., Yu Z., Hou J., Yan H., Yuan W. (2025). USP10 Inhibits Ferroptosis via Deubiquinating POLR2A in Head and Neck Squamous Cell Carcinoma. Adv. Sci..

[117] Jacq X., Kemp M., Martin N.M.B., Jackson S.P. (2013). Deubiquitylating Enzymes and DNA Damage Response Pathways. Cell Biochem. Biophys..

[118] Bomberger J.M., Barnaby R.L., Stanton B.A. (2009). The Deubiquitinating Enzyme USP10 Regulates the Post-Endocytic Sorting of Cystic Fibrosis Transmembrane Conductance Regulator in Airway Epithelial Cells. J. Biol. Chem..

[119] Lu C., Ning Z., Wang A., Chen D., Liu X., Xia T., Tekcham D.S., Wang W., Li T., Liu X. (2018). USP10 Suppresses Tumor Progression by Inhibiting mTOR Activation in Hepatocellular Carcinoma. Cancer Lett..

[120] Nakayama M., Hong C.P., Oshima H., Sakai E., Kim S.-J., Oshima M. (2020). Loss of Wild-Type P53 Promotes Mutant P53-Driven Metastasis through Acquisition of Survival and Tumor-Initiating Properties. Nat. Commun..

[121] Kubaichuk K., Seitz T., Bergmann U., Glumoff V., Mennerich D., Kietzmann T. (2024). Ubiquitin-Specific Protease 10 Determines Colorectal Cancer Outcome by Modulating Epidermal Growth Factor Signaling via Inositol Polyphosphate-4-Phosphatase Type IIB. Oncogenesis.

[122] Vogelstein B., Lane D., Levine A.J. (2000). Surfing the P53 Network. Nature.

[123] Li D.M., Sun H. (1998). PTEN/MMAC1/TEP1 Suppresses the Tumorigenicity and Induces G1 Cell Cycle Arrest in Human Glioblastoma Cells. Proc. Natl. Acad. Sci. USA.

[124] Lee J.T., Shan J., Zhong J., Li M., Zhou B., Zhou A., Parsons R., Gu W. (2013). RFP-Mediated Ubiquitination of PTEN Modulates Its Effect on AKT Activation. Cell Res..

[125] Mushajiang M., Li Y., Sun Z., Liu J., Zhang L., Wang Z. (2024). USP10 Alleviates Nε-Carboxymethyl-Lysine-Induced Vascular Calcification and Atherogenesis in Diabetes Mellitus by Promoting AMPK Activation. Cell Signal..

[126] Sun J., Li T., Zhao Y., Huang L., Sun H., Wu H., Jiang X. (2018). USP10 Inhibits Lung Cancer Cell Growth and Invasion by Upregulating PTEN. Mol. Cell Biochem..

[127] Muller P.A.J., Vousden K.H. (2014). Mutant P53 in Cancer: New Functions and Therapeutic Opportunities. Cancer Cell.

[128] Guo K., Ma Z., Zhang Y., Han L., Shao C., Feng Y., Gao F., Di S., Zhang Z., Zhang J. (2022). HDAC7 Promotes NSCLC Proliferation and Metastasis via Stabilization by Deubiquitinase USP10 and Activation of β-Catenin-FGF18 Pathway. J. Exp. Clin. Cancer Res..

[129] Anusewicz D., Orzechowska M., Bednarek A.K. (2021). Notch Signaling Pathway in Cancer-Review with Bioinformatic Analysis. Cancers.

[130] Krossa I., Strub T., Martel A., Nahon-Esteve S., Lassalle S., Hofman P., Baillif S., Ballotti R., Bertolotto C. (2022). Recent Advances in Understanding the Role of HES6 in Cancers. Theranostics.

[131] Shi Q., Xue C., Zeng Y., Yuan X., Chu Q., Jiang S., Wang J., Zhang Y., Zhu D., Li L. (2024). Notch Signaling Pathway in Cancer: From Mechanistic Insights to Targeted Therapies. Signal Transduct. Target. Ther..

[132] Thrash H.L., Pendergast A.M. (2023). Multi-Functional Regulation by YAP/TAZ Signaling Networks in Tumor Progression and Metastasis. Cancers.

[133] Cao J., Wang X., Wang S., Chen Z., Tang J. (2023). Stabilization of Estrogen Receptor α by USP37 Contributes to the Progression of Breast Cancer. Cancer Sci..

[134] Niu Z., Li X., Feng S., Huang Q., Zhuang T., Yan C., Qian H., Ding Y., Zhu J., Xu W. (2020). The Deubiquitinating Enzyme USP1 Modulates ERα and Modulates Breast Cancer Progression. J. Cancer.

[135] Dindi U.M.R., Al-Ghamdi S., Alrudian N.A., Dayel S.B., Abuderman A.A., Saad Alqahtani M., Bahakim N.O., Ramesh T., Vilwanathan R. (2023). Ameliorative Inhibition of Sirtuin 6 by Imidazole Derivative Triggers Oxidative Stress-Mediated Apoptosis Associated with Nrf2/Keap1 Signaling in Non-Small Cell Lung Cancer Cell Lines. Front. Pharmacol..

[136] Haley J.A., Ruiz C.F., Montal E.D., Wang D., Haley J.D., Girnun G.D. (2019). Decoupling of Nrf2 Expression Promotes Mesenchymal State Maintenance in Non-Small Cell Lung Cancer. Cancers.

[137] Liu X., Ren S., Li Z., Hao D., Zhao X., Zhang Z., Liu D. (2023). Sirt6 Mediates Antioxidative Functions by Increasing Nrf2 Abundance. Exp. Cell Res..

[138] Fu W., Xiao Z., Chen Y., Pei J., Sun Y., Zhang Z., Wu H., Pei Y., Wei S., Wang Y. (2023). Molecular Integrative Study on Interaction Domains of Nuclear Factor Erythroid 2-Related Factor 2 with Sirtuin 6. Biochimie.

[139] Khan A.Q., Siveen K.S., Prabhu K.S., Kuttikrishnan S., Akhtar S., Shaar A., Raza A., Mraiche F., Dermime S., Uddin S. (2018). Curcumin-Mediated Degradation of S-Phase Kinase Protein 2 Induces Cytotoxic Effects in Human Papillomavirus-Positive and Negative Squamous Carcinoma Cells. Front. Oncol..

[140] Zhao B., Zong G., Xie Y., Li J., Wang H., Bian E. (2015). Downregulation of Ubiquitin-Associated Protein 2-like with a Short Hairpin RNA Inhibits Human Glioma Cell Growth In Vitro. Int. J. Mol. Med..

[141] Ding L., Li R., Sun R., Zhou Y., Zhou Y., Han X., Cui Y., Wang W., Lv Q., Bai J. (2017). S-Phase Kinase-Associated Protein 2 Promotes Cell Growth and Motility in Osteosarcoma Cells. Cell Cycle.

[142] Ding L., Li R., Han X., Zhou Y., Zhang H., Cui Y., Wang W., Bai J. (2017). Inhibition of Skp2 Suppresses the Proliferation and Invasion of Osteosarcoma Cells. Oncol. Rep..

[143] Jang W., Kim T., Koo J.S., Kim S.-K., Lim D.-S. (2017). Mechanical Cue-Induced YAP Instructs Skp2-Dependent Cell Cycle Exit and Oncogenic Signaling. EMBO J..

[144] Xia X., Liu X., Chai R., Xu Q., Luo Z., Gu J., Jin Y., Hu T., Yu C., Du B. (2021). USP10 Exacerbates Neointima Formation by Stabilizing Skp2 Protein in Vascular Smooth Muscle Cells. J. Biol. Chem..

[145] Zeng Y., Zhu J., Shen D., Qin H., Lei Z., Li W., Huang J.-A., Liu Z. (2016). Repression of Smad4 by miR-205 Moderates TGF-β-Induced Epithelial-Mesenchymal Transition in A549 Cell Lines. Int. J. Oncol..

[146] Huang M.-L., Zou Y., Yang R., Jiang Y., Sheng J.-F., Han J.-B., Kong Y.-G., Tao Z.-Z., Chen S.-M. (2019). Placenta Specific 8 Gene Induces Epithelial-Mesenchymal Transition of Nasopharyngeal Carcinoma Cells via the TGF-β/Smad Pathway. Exp. Cell Res..

[147] Spencer-Smith R., Morrison D.K. (2024). Regulation of RAF Family Kinases: New Insights from Recent Structural and Biochemical Studies. Biochem. Soc. Trans..

[148] Bekele R.T., Samant A.S., Nassar A.H., So J., Garcia E.P., Curran C.R., Hwang J.H., Mayhew D.L., Nag A., Thorner A.R. (2021). RAF1 Amplification Drives a Subset of Bladder Tumors and Confers Sensitivity to MAPK-Directed Therapeutics. J. Clin. Investig..

[149] Clark-Garvey S., Kim W.Y. (2021). RAF1 Amplification: An Exemplar of MAPK Pathway Activation in Urothelial Carcinoma. J. Clin. Investig..

[150] Lin Z., Yang H., Tan C., Li J., Liu Z., Quan Q., Kong S., Ye J., Gao B., Fang D. (2013). USP10 Antagonizes C-Myc Transcriptional Activation through SIRT6 Stabilization to Suppress Tumor Formation. Cell Rep..

[151] Sun J., Xiang Q., Ding D., Yan N. (2023). USP10 Suppresses ABCG2-Induced Malignant Characteristics of Doxorubicin-Resistant Thyroid Cancer by Inhibiting PI3K/AKT Pathway. J. Bioenerg. Biomembr..

[152] Wang X., Xia S., Li H., Wang X., Li C., Chao Y., Zhang L., Han C. (2020). The Deubiquitinase USP10 Regulates KLF4 Stability and Suppresses Lung Tumorigenesis. Cell Death Differ..

[153] Pang D., Yu Y., Zhao B., Huang J., Cui Y., Li T., Li C., Shang H. (2024). The Long Non-Coding RNA NR3C2-8:1 Promotes P53-Mediated Apoptosis through the miR-129-5p/USP10 Axis in Amyotrophic Lateral Sclerosis. Mol. Neurobiol..

[154] Ko A., Han S.Y., Choi C.H., Cho H., Lee M.-S., Kim S.-Y., Song J.S., Hong K.-M., Lee H.-W., Hewitt S.M. (2018). Oncogene-Induced Senescence Mediated by c-Myc Requires USP10 Dependent Deubiquitination and Stabilization of p14ARF. Cell Death Differ..

[155] Xin S.-L., Pan X.-L., Xu X.-Y., Yu Y.-Y. (2023). USP10 Alleviates Palmitic Acid-Induced Steatosis through Autophagy in HepG2 Cells. J. Clin. Transl. Hepatol..

[156] Cheng X., Xu X., Chen D., Zhao F., Wang W. (2019). Therapeutic Potential of Targeting the Wnt/β-Catenin Signaling Pathway in Colorectal Cancer. Biomed. Pharmacother..

[157] Wang Y., Liu J., Zheng S., Cao L., Li Y., Sheng R. (2023). The Deubiquitinase USP10 Mediates Crosstalk between the LKB1/AMPK Axis and Wnt/β-Catenin Signaling in Cancer. FEBS Lett..

[158] Liu J., Zhang S., Cao L., Zhang N., Guo Q., Zou Y., Yang R., Dong S., Zheng L., Xiao Y. (2025). The Deubiquitination-PARylation Positive Feedback Loop of the USP10-PARP1 Axis Promotes DNA Damage Repair and Affects Therapeutic Efficacy of PARP1 Inhibitor. Oncogene.

[159] Reissland M., Hartmann O., Tauch S., Bugter J.M., Prieto-Garcia C., Schulte C., Loebbert S., Solvie D., Bitman-Lotan E., Narain A. (2024). USP10 Drives Cancer Stemness and Enables Super-Competitor Signalling in Colorectal Cancer. Oncogene.

[160] Chen T., Ashwood L.M., Kondrashova O., Strasser A., Kelly G., Sutherland K.D. (2025). Breathing New Insights into the Role of Mutant P53 in Lung Cancer. Oncogene.

[161] Zhu G., Pan C., Bei J.-X., Li B., Liang C., Xu Y., Fu X. (2020). Mutant P53 in Cancer Progression and Targeted Therapies. Front. Oncol..

[162] Xin X., Li Z., Yan X., Liu T., Li Z., Chen Z., Yan X., Zeng F., Hou L., Zhang J. (2024). Hepatocyte-Specific Smad4 Deficiency Inhibits Hepatocarcinogenesis by Promoting CXCL10/CXCR3-Dependent CD8+- T Cell-Mediated Anti-Tumor Immunity. Theranostics.

[163] Han Y., Kong W., Shang Q., Liu Y., Ni X., Yang L., Lei J. (2025). Discovery of Targeting USP10-Mediated Proline Metabolism Arrangement to Inhibit Hepatocellular Carcinoma Progression. Biochem. Pharmacol..

[164] Puissant A., Fenouille N., Alexe G., Pikman Y., Bassil C.F., Mehta S., Du J., Kazi J.U., Luciano F., Rönnstrand L. (2014). SYK Is a Critical Regulator of FLT3 in Acute Myeloid Leukemia. Cancer Cell.

[165] Zangooie A., Tavoosi S., Arabhosseini M., Halimi A., Zangooie H., Baghsheikhi A.H., Rahgozar S., Ahmadvand M., Jarrahi A.M., Salehi Z. (2024). Ubiquitin-Specific Proteases (USPs) in Leukemia: A Systematic Review. BMC Cancer.

[166] Zhao J., Guo J., Wang Y., Ma Q., Shi Y., Cheng F., Lu Q., Fu W., Ouyang G., Zhang J. (2022). Research Progress of DUB Enzyme in Hepatocellular Carcinoma. Front. Oncol..

[167] Liao Y., Guo Z., Xia X., Liu Y., Huang C., Jiang L., Wang X., Liu J., Huang H. (2019). Inhibition of EGFR Signaling with Spautin-1 Represents a Novel Therapeutics for Prostate Cancer. J. Exp. Clin. Cancer Res..

[168] Yu M., Fang Z.-X., Wang W.-W., Zhang Y., Bu Z.-L., Liu M., Xiao X.-H., Zhang Z.-L., Zhang X.-M., Cao Y. (2021). Wu-5, a Novel USP10 Inhibitor, Enhances Crenolanib-Induced FLT3-ITD-Positive AML Cell Death via Inhibiting FLT3 and AMPK Pathways. Acta Pharmacol. Sin..

